# Conductance in a bis-terpyridine based single molecular breadboard circuit†
†Electronic supplementary information (ESI) available. See DOI: 10.1039/c6sc03204d


**DOI:** 10.1039/c6sc03204d

**Published:** 2016-11-03

**Authors:** Charu Seth, Veerabhadrarao Kaliginedi, Sankarrao Suravarapu, David Reber, Wenjing Hong, Thomas Wandlowski, Frédéric Lafolet, Peter Broekmann, Guy Royal, Ravindra Venkatramani

**Affiliations:** a Department of Chemical Sciences , Tata Institute of Fundamental Research , Homi Bhabha Road, Colaba , Mumbai 400 005 , India . Email: ravi.venkatramani@tifr.res.in; b Department of Chemistry and Biochemistry , University of Bern , Freiestrasse 3, CH-3012 , Bern , Switzerland . Email: veera.kaliginedi@epfl.ch; c Department of Chemical and Biochemical Engineering , College of Chemistry and Chemical Engineering , Xiamen University , Xiamen 361005 , China; d Université Grenoble Alpes , Département de Chimie Moléculaire , UMR CNRS-5250 , Institut de Chimie Moléculaire de Grenoble , FR CNRS-2607 , BP 53 , 38041 Grenoble Cedex 9 , France . Email: guy.royal@univ-grenoble-alpes.fr

## Abstract

Controlling charge flow in single molecule circuits with multiple electrical contacts and conductance pathways is a much sought after goal in molecular electronics. In this joint experimental and theoretical study, we advance the possibility of creating single molecule breadboard circuits through an analysis of the conductance of a bis-terpyridine based molecule (**TP1**). The **TP1** molecule can adopt multiple conformations through relative rotations of 7 aromatic rings and can attach to electrodes in 61 possible single and multi-terminal configurations through 6 pyridyl groups. Despite this complexity, we show that it is possible to extract well defined conductance features for the **TP1** breadboard and assign them rigorously to the underlying constituent circuits. Mechanically controllable break-junction (MCBJ) experiments on the **TP1** molecular breadboard show an unprecedented 4 conductance states spanning a range 10 ^–2^ *G*_0_ to 10 ^–7^ *G*_0_. Quantitative theoretical examination of the conductance of **TP1** reveals that combinations of 5 types of single terminal 2–5 ring subcircuits are accessed as a function of electrode separation to produce the distinct conductance steps observed in the MCBJ experiments. We estimate the absolute conductance for each single terminal subcircuit and its percentage contribution to the 4 experimentally observed conductance states. We also provide a detailed analysis of the role of quantum interference and thermal fluctuations in modulating conductance within the subcircuits of the **TP1** molecular breadboard. Finally, we discuss the possible development of molecular circuit theory and experimental advances necessary for mapping conductance through complex single molecular breadboard circuits in terms of their constituent subcircuits.

## Abstract 2

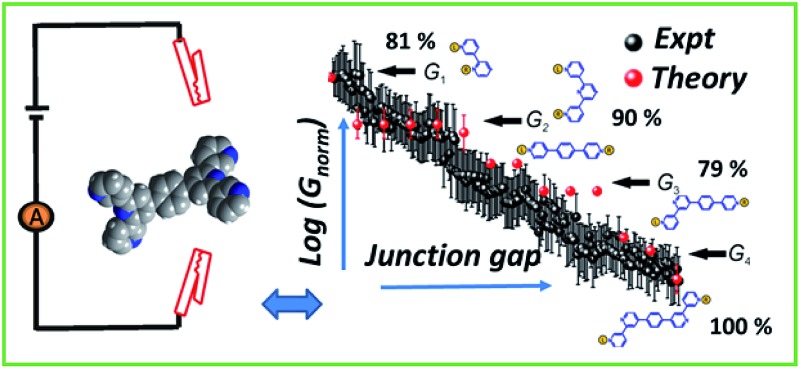
We study conductance in a molecular breadboard junction accommodating up to 61 circuits and demonstrate switching between 4 conductance states.

## Introduction

The central goal in molecular electronics is to develop molecules as active electronic circuit elements.^1,2^ Towards this end, experimental techniques based on a Scanning Tunneling Microscope Break Junction (STM-BJ)^3^ or Mechanically Controllable Break Junction (MCBJ) have been developed to study molecular charge transport properties at a single molecule level.^4–8^ In STM-BJ/MCBJ, single molecules are contacted by two electrodes to form a metal–molecule–metal junction. Under the application of a bias voltage, the current flowing through the molecule, from one electrode to the other is sensitive to the molecular structure (length, conjugation pattern and conformation),^9,10^ the chemical nature of the linker which connects the molecule to the electrode,^6,11–13^ the relative position of linkers attached to the molecular core,^14,15^ the binding geometry of molecule between the electrodes,^16–18^ and the effect of external triggers on molecular structure and environment.^2,6,19–22^ Thus, these experimental techniques when combined with charge transport theory present a unique opportunity to create dynamic circuits with a library of molecules, which can act like wires,^9,23–25^ switches^26,27^ and diodes.^21,28,29^

**Fig. 1:**
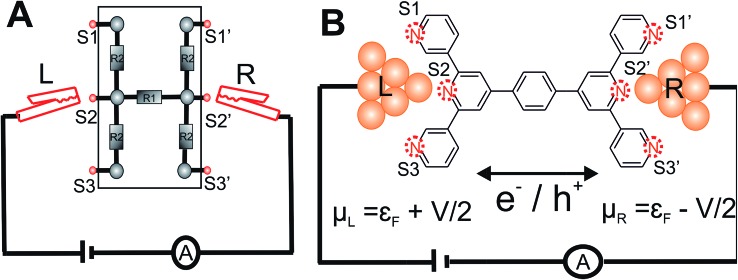
Enumeration of circuits within **TP1** molecular breadboard: (A) schematic of a conventional electrical breadboard. For the breadboard depicted, multiple circuits of varying complexity can be created by throwing different permutations and combinations of the switches (S1-3, S1′-3′) (B) schematic of molecular breadboard junction formed for the **TP1** molecule within a break-junction type setup. The **TP1** molecular breadboard offers multiple circuits defined by the different contacts (S1-3, S1′-3′) made at nitrogen atoms by two electrodes.

In conventional electrical and electronics engineering, solderless breadboard scaffolds are routinely used for quickly creating/testing a range of electrical circuits of varied complexity (Fig. 1A). Adapting the concept of breadboard on a molecular scale to prototype molecular circuits is an attractive idea. Aided by developments in synthetic methodologies, chemists are now able to synthesize large molecules incorporating different functional groups which in principle can form active or passive circuit elements. In order to develop the concept of a molecular breadboard circuit within a break junction framework, the choice of the chemical linker group is very important. The linker provides reliable attachment of molecules to the electrodes, determines the electronic coupling of the molecular core with the electrodes, and controls the range of relative orientations of the molecule with respect to the electrode. Experimentally, the conductance of organic molecules utilizing a number of anchoring groups have been compared including benzothiophene (–BT), pyridyl (–PY), amino (–NH_2_), thiol (–SH), isothiocyanide (–SCN), cyanide (–CN), nitro (–NO_2_), carboxylic acid (–COOH), dimethyl phosphine (–PMe_2_), methyl sulphide (–SMe), hydroxyl (–OH), and carbodithiolate based linkers.^6,11–13,30^ These studies have helped deconvolute the effect of the linkers from that of the molecular core on the single molecular junction conductance. Several experimental and theoretical attempts have been made to understand and/or predict the possible quantum interference patterns which govern the conductance properties of conductance pathways across the molecular core.^14,31–37^ These studies compared the relative conductance of molecules which differed in terms of either the chemical structure of the molecular core or the placement of anchoring groups within the molecule. In this context, the introduction of molecular breadboard circuits would be a significant advance, enabling the comparison of multiple conductance pathways within a single molecular scaffold. Indeed, steps have been taken towards realizing multiple circuits within a single molecular junction. Kiguchi and co-workers have recently demonstrated a conductance switch based on control of the molecule-electrode contacts within a quarterthiopene molecular junction with 4 thiophene anchoring groups.^17^ The authors captured 3 distinct conductance states and assigned it to bithiophene, trithiophene, and quarterthiophene circuits.^17^ Miguel and co-workers recently demonstrated 2 stable conductance pathways within a *para*-oligophenylene ethylene (*p*-OPE) molecular scaffold. By substituting the central benzene ring with sulfur and nitrogen heterocycles, the authors were able to demonstrate a new high conductance channel formed by the linking of the central pyrimidine ring to the electrodes.^18^ More recently, Kiguchi and co-workers have also shown the existence of 3 conductance states within a tripyridyl–triazine molecular junctions.^38^ In the studies above ^17,18,38^ single circuits were assigned to each conductance state observed in the experiments and upto 3 circuits across the molecular scaffold were proposed to be accessible by the experiments.

Here, we present the MCBJ implementation of a terpyridine based molecular breadboard (4′,4′′′′-(1,4-phenylene)bis(3,2′:6′,3′′-terpyridine), **TP1**), wherein multiple electrode anchoring pyridyl groups create 61 possible breadboard circuits within a single molecular scaffold (Fig. 1B). Further, each circuit has multiple thermally accessible conformations due to the molecular torsional flexibility. We experimentally capture 4 distinct conductance states for **TP1** spanning 5 orders of conductance range as a function of electrode separation. A quantitative computational analysis of conductance and structures of over 1000 molecular circuits embedded within the **TP1** breadboard reveals that the switching between different conductance states is controlled by accessing combinations of single and multi-terminal circuits within the breadboard. We find that the flexibility in molecular geometry imparted by the ring rotation degrees of freedom leads to less than one order of magnitude variation in the overall molecular conductance of **TP1**. Based on our analysis we are able to assign specific combinations of single terminal 2–5 ring circuits to each experimentally observed conductance state, estimate their percentage contribution to each state and extract their absolute conductance values. The role of quantum interference effects, thermal fluctuations, and possibility of formulating circuit rules for the **TP1** breadboard are discussed.

## Methods

### Synthesis of **TP1** and associated molecules

Details of the synthesis and characterization of all molecules examined in this manuscript are provided in Section S.1 of ESI.†

### Molecular junction conductance measurements

Single molecular conductance measurements were performed by employing home built mechanically controllable break junction set up. Further technical details of the MCBJ setup and data analysis procedures were reported in our previous publications.^9,14,39^ All the measurements were done in solution at room temperature. We provide more details in Section S.2 of the ESI.†

### Theory

We use the framework of Non-Equilibrium Greens Functions (NEGF) to compute single molecule conductance of the **TP1** molecule. The NEGF calculations are based on an INDO/s Hamiltonian and DFT optimized geometries of the molecule. This computational framework has been described in detail previously and shown to be effective in several previous studies of charge transport properties of small organic molecules.^40–42^ We outline the procedure for studying **TP1** below. A more detailed description is provided in Sections S.3–S.5 of ESI.†

#### Electronic structure calculations

The **TP1** molecule is expected to exhibit significant conformational flexibility in terms of ring rotation. We therefore considered multiple molecular geometries which differ in terms of their relative orientations of the pyridine rings in our study. Different conformations of **TP1** were manually drawn wherein each aromatic ring was allowed to adopt one of two conformations, either in plane or orthogonal, relative to its neighboring rings. This procedure resulting in 24 distinct starting geometries, which were optimized in Gaussian 09 using DFT with a B3LYP exchange correlation functional and a 6-31G* basis set.^43^ The optimization yielded 18 unique geometries (detailed results provided in Section S.3 of ESI†). For each optimized geometry we computed the electronic structure of the molecule using the semi-empirical INDO/s Hamiltonian,^44^ as implemented in the CNDO program.^45^ We note that while the optimization procedure yields local minima, the diverse starting conformations ensure that the full range of ring conjugation (and associated variability in conductance) is captured for the **TP1** breadboard potential energy landscape. Further, as all conformations are thermally accessible at room temperature, additional sampling through molecular dynamics simulations around each local minimum is expected to refine (by producing more realistic broadenings for conductance histograms) but not alter any of the conclusions presented here.

#### Computation of the molecular conductance

The conductance for each of the 61 molecular circuits enumerated in Table 1 and their 18 optimized geometries (1098 circuit calculations) was computed using the NEGF framework;^46^ we assumed that the **TP1** molecule is connected to the electrodes through the nitrogen atoms of the flanking terpyridine arms (Fig. 1A). We adopt the weak coupling limit, discussed extensively in Xing *et al.*,^41^ wherein the electrode atoms are not explicitly modelled. Instead, the effect of the electrodes is introduced through the broadening matrix (*Γ*) in the atomic basis with non-zero elements corresponding to the coupling of specific atoms to the electrode (eqn (1)). We assume that only nitrogen atoms are electronically coupled to the electrodes (*Γ*_N_ = 0.1 eV). However, nitrogen atoms on the peripheral pyridine rings of the terpyridine units are more accessible to the electrodes than those on the central pyridine rings. We thus constructed a model wherein the broadening matrix includes different electrode electronic couplings for peripheral (end pyridine rings) and core (central pyridine rings) nitrogen atoms on the terpyridine units of **TP1**:1
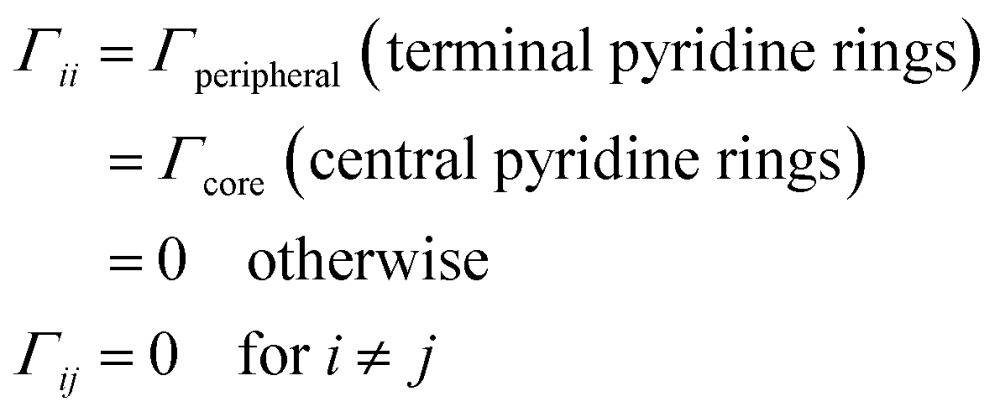
here *Γ*_core_ is exponentially screened relative to *Γ*_peripheral_: *Γ*_peripheral_/*Γ*_core_ = exp(*β × d*_eff_), where *β* = 3.0 Å^–1^ is the decay of the electronic coupling through vacuum. The effective screening length *d*_eff_ was defined as the distance between the central (core) nitrogen atom and the centre of mass of the peripheral nitrogen atoms for each terpyridine arm of **TP1**. From a structural analysis of the 18 optimized geometries of **TP1** (see Section S.7 of ESI†), we estimated *d*_eff_ ∼ 1.6 Å, leading to an electronic coupling attenuation ratio *Γ*_peripheral_/*Γ*_core_ = 116. Independently, we also computed the conductance of **TP1** for different *Γ*_peripheral_/*Γ*_core_ ratios ranging from 1–1000 and examined the fit of the computed conductance to the experimental data. The analysis reveals that a model with *Γ*_peripheral_/*Γ*_core_ ∼ 100 yields the best fits to the experimental data (Section S.7 of ESI†) independently corroborating the electronic coupling attenuation obtained from the geometry analysis of **TP1**. The results presented in the main manuscript are for *Γ*_peripheral_/*Γ*_core_ = 116 (data for other ratios are provided in the ESI†). We neglect the real part of the self-energy in our calculations. In the linear response regime, the conductance is given by the Landauer expression:2
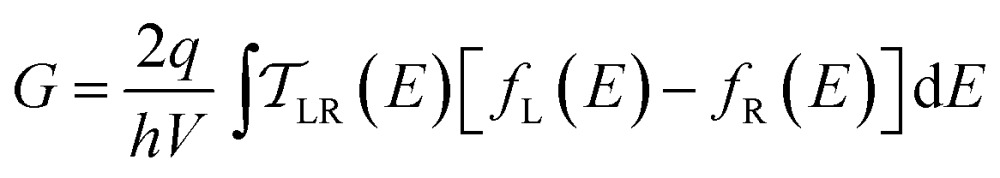
where the transmission function 
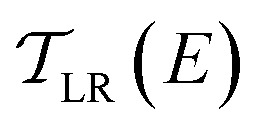
 is computed from the INDO/S Hamiltonian as described previously and in Section S.5 of ESI.† The Fermi functions *f*_L/R_ define the electron occupancy based on the chemical potentials *μ*_L/R_ (Fig. 1B) of left and right electrode:3
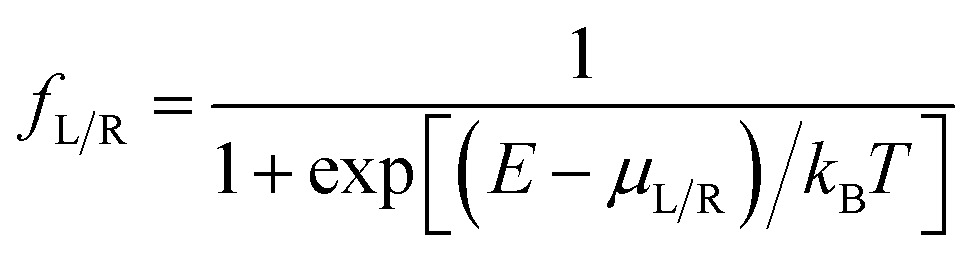
where *k*_B_ is the Boltzmann constant and temperature *T* = 300 K. The Fermi energy *E*_F_ is set to –5.1 eV, corresponding to the work function of gold, and *V* is the applied potential bias (taken as 100 mV in the calculations here). The potential drop is assumed to be symmetric across the electrodes. The small biases applied here are not expected to cause significant changes in molecular geometry or energies, and no potential drop is assumed across the molecule. For the INDO/s band gaps calculated for **TP1** geometries, the electrode Fermi level (set to –5.1 eV) lies closer to the HOMO (hole transport barrier of ∼2.9 eV) than the LUMO. DFT calculated band gaps are lower with the electrode Fermi level again lying closer to the HOMO (hole transport barrier of ∼1.4 eV) than the LUMO. However, recent experiments on bis-terpyridine poly-*p*-phenyl molecules and molecules with pyridyl anchors have suggested that transport in these systems is in fact electron dominated.^47,48^ Thus, we also carried out calculations in the electron dominated regime by setting the Fermi level to 2.9 eV below the LUMO (Section S.8 of ESI†). We find that the conductance features of the **TP1** breadboard, and relative conductance trends for various circuits are insensitive to whether the transport takes place in the hole dominated or electron dominated regime (see Section S.9 of ESI†). For the large barriers (>1 eV) and small molecular bridge lengths (<20 Å) for the **TP1** system studied here, charge transport takes place *via* tunneling^49^ and the conductance is dominated by the contribution at the Fermi energy:^42^
4
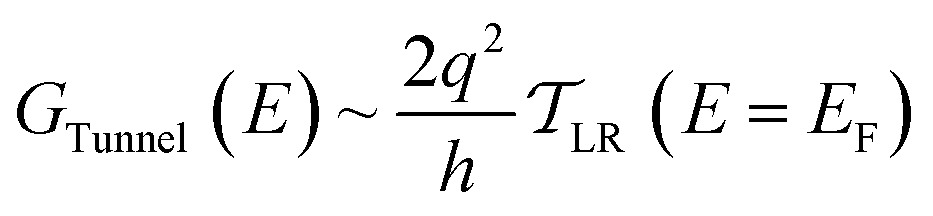



**Table 1:** We enumerate all contact configurations wherein the left (L) and right (R) electrodes each contact distinct sets of nitrogen atoms (S1-3, S1′-3′) on each of the two **TP1** arms. These include symmetric single (1_L_–1_R_), double (2_L_–2_R_), and triple (3_L_–3_R_) contact configurations, as well as asymmetric contact configurations (*M*_L_–*N*_R_; *M* ≠ *N*), where *M*_L/R_ and *N*_L/R_ represent number of atoms contacting the L/R electrodes. We assume that multiple atoms across the two terpyridine arms cannot be contacted by a single electrode. Examples of single/multi-terminal circuits and decomposition of multi-terminal circuits into constituent single terminal circuits are shown in the third column. *N*-Ring refers to the number of aromatic rings in the single terminal circuit. See also Fig. 2 for depictions of these circuits

Contact configuration	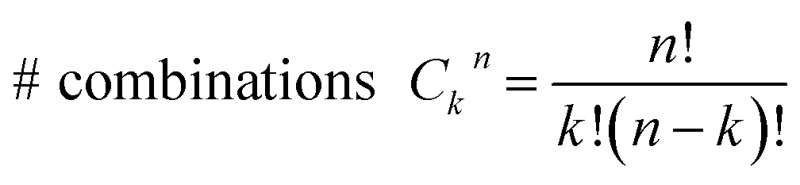	Examples
1_L_–1_R_	(*C*_1_^6^ × *C*_1_^5^)/2 = 15	2-ring: L(S1′)–R(S2′)
		3-ring: L(S1)–R(S1′), L(S1)–R(S3)
1_L_–2_R_ or 2_L_–1_R_	*C*_2_^3^ × *C*_1_^4^ = 12 (24 total)	2-ring + 3-ring: L(S1, S2)–R(S3)
		2 × 2-ring: L(S1, S3)–R(S2)
		4-ring + 3-ring: L(S1, S3)–R(S2′)
1_L_–3_R_ or 3_L_–1_R_	*C*_1_^3^ × *C*_3_^3^ = 3 (6 total)	4-ring + 2 × 5-ring: L(S1)–R(S1′, S2′, S3′)
		3-ring + 4-ring + 5-ring: L(S1, S2, S3)–R(S2′)
2_L_–2_R_	*C*_2_^3^ × *C*_2_^3^ = 9	4 × 5-ring: L(S1, S3)–R(S1′, S3′)
		3-ring + 2 × 4-ring + 5-ring: L(S1, S2)–R(S1′, S2′)
2_L_–3_R_ or 3_L_–2_R_	*C*_2_^3^ × *C*_3_^3^ = 3 (6 total)	4 × 5-ring + 2 × 4-ring: L(S1, S3)–R(S1′, S2′, S3′)
		3-ring + 3 × 4-ring + 2 × 5-ring: L(S1, S2)–R(S1′, S2′, S3′)
3_L_–3_R_	*C*_3_^3^ × *C*_3_^3^ = 1	3-ring + 4-ring + 5-ring: L(S1, S2, S3)–R(S1′, S2′, S3′)

The computational framework described here is not expected to reproduce absolute values of the experimental conductance due to the lack of knowledge of the exact form of the molecule-electrode coupling, uncertainty in the placement of the electrode Fermi level with respect to the molecular energy levels, and neglect of solvent in our models. However, as demonstrated previously, the framework can reliably reproduce relative trends in charge transport properties.^40–42^ Our calculations are thus expected to capture relative trends in the conductance of the circuits within the **TPI** molecular breadboard as a function of conformation and molecule-electrode contact configurations.

#### Critical discussion on model assumptions

Our computational protocol considers multiple thermally accessible **TP1** geometries (*vide supra*) that the single molecule break-junction experiment might sample in individual traces to build the conductance statistics for the junction. In these calculations, the conductance is primarily averaged over dihedral degrees of freedom and neglects inelastic contributions to charge transport. Inelastic contributions arise due to the coupling of the electronic transport to molecular vibrational degrees of freedom and become prominent when the dwell/transit times of the electron inside the molecular barrier become comparable to the vibrational timescale. For the **TP1** molecule, the length of sub-circuits lies between 4–16 Å and barriers for tunneling are >1 eV. Thus, for these systems the Landauer–Buttiker barrier transit time^50^ is of the order of a few fs and shorter than the timescale of vibrational motions. The fastest motions which might compete are the vibrations of bonds between hydrogens and heavy atoms (>10 fs timescale), which do not significantly influence the dominant π transport channels in these systems. These considerations allow us to neglect inelastic contributions to charge transport in our calculations.

Our calculations neglect electronic correlations for metal electrodes which couple to the molecules. Such correlations can lead to interesting interference effects boosting or suppressing multi-terminal circuit currents relative to that obtained by a simple addition of currents from the constituent single terminal circuits.^51,52^ The neglect of correlated electron injection thus allows a simple decomposition of multi-terminal circuits into a superposition of single terminal circuits. Several considerations justify our choice of neglecting electron correlation between electrodes coupling to the **TP1** molecule in the present study:

(1) Neither the gold coordination sphere around the nitrogen atoms of **TP1** nor the shape of electrode tips interacting with the molecule is precisely known. Further, for the **TP1** molecule the pyridine ring rotational degrees of freedom alter the relative positions of nitrogen atoms (see Fig. S5A†), such that even the closest nitrogen atoms located on adjacent pyridine rings (4–5 Å separation) may interact with distinct clusters of gold atoms.

(2) It is possible, of course, that nitrogen atoms on adjacent pyridine rings may share their gold coordination sphere for some **TP1** geometries. However, in this case, the multi-terminal circuits are composed of asymmetric (in terms of number of aromatic rings) single terminal circuits with significantly different conductance values. For instance, in Fig. 1B the multi-terminal circuit L(S1, S2)–R(S1′, S2′) is composed of 3-ring (L(S2)–R(S2′)), 4-ring (L(S1)–R(S2′)/L(S2)–R(S1′)), and 5-ring (L(S1)–R(S1′)) circuits. Since such asymmetric circuits have very different conductance values (differing by 1–2 orders of magnitude; see Table 2 last row) any interference effects arising from correlated electronic injection at positions S1 and S2 would be negligible.

(3) Finally, for multi-terminal circuits with symmetric single terminal conductance pathways such as L(S1, S3)–R(S1′, S3′) or L(S1, S3)–R(S2), the relevant terminal nitrogen atoms (S1 and S3) are well separated (∼8 Å), and a model assuming independent electronic coupling at these positions is reasonable.

## Results

### Enumeration of the circuits in the **TP1** molecular breadboard

**Fig. 2:**
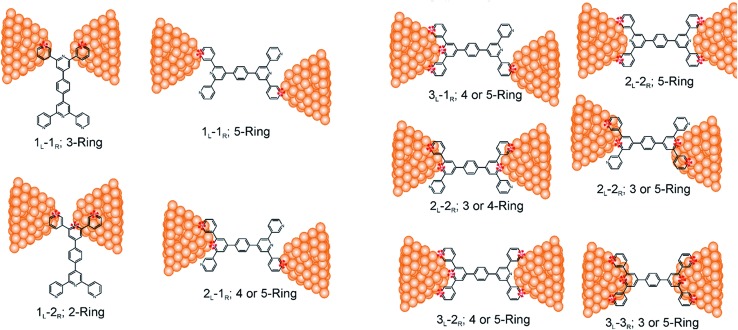
Examples of single and multi-terminal circuits for the **TP1** that can potentially form in break junction experiments. Nitrogen atoms which are interacting with electrode are indicated in red color.

Consider a conventional macroscopic electrical breadboard circuit as shown in Fig. 1A, where different circuits can be created by contacting a pair of sockets (from S1, S2, S3, S1′, S2′, S3′) with two alligator clips. The number of possible 2 terminal circuits thus formed is *C*62 = 15 (where *C*_*k*_^*n*^ = *n*!/*k*!(*n* – *k*)! denotes the number of ways to pick *k* sockets from a set of *n* sockets). If we allow each of the two alligator clips to contact more than one socket, the breadboard can potentially offer up to 301 single and multi-terminal circuits (see Section S.4 in ESI† for detailed enumeration). Analogously, to enumerate circuits within the **TP1** molecular breadboard (Fig. 1B), we consider the basic assumptions of two left (L) and right (R) contacting electrodes, circuits with symmetric transmission (*I*_L→R_(*V*_bias_) = *I*_R→L_(*V*_bias_)), and distinct sets of nitrogen atoms contacted by each electrode. Expressed in terms of the number of contacts (*M*, *N*) made by the two (L/R) electrodes, contact configurations (*M*_L_–*N*_R_, *M*, *N* = 1, 2, 3) can be symmetric (*M* = *N*) or asymmetric (*M* ≠ *N*). Here, we assume that a single electrode cannot contact pyridyl groups on both terpyridine arms of **TP1** simultaneously. Further, in break junction experiments (*e.g.* MCBJ) the smallest electrode separation formed in a MCBJ experiment after breaking gold–gold contact (snap-back distance) is estimated to be ∼0.4–0.6 nm,^9,11,12^ which allows both symmetric and asymmetric circuits within each of the terpyridine arms of **TP1**. Under these constraints, 61 distinct circuits can be formed across the **TP1** breadboard, which are enumerated in Table 1. We note that multi-terminal circuits can be decomposed into constituent single terminal circuits. For instance, the 1_L_–2_R_/2_L_–1_R_ circuits can be decomposed (see Fig. 2) into two single terminal circuits which may be comprised of: (1) one 2-ring and one 3-ring circuit, (2) two 2-ring circuits, (3) one 4-ring and one 3-ring circuit, or (4) one 4-ring and one 5-ring circuit. These examples and other single and multi-terminal connections that are possible within a two electrode junction are summarized in Table 1 and Fig. 2. We will comment on the relationship between the currents from multi-terminal circuits and that from their single terminal constituent circuits in later subsections which discuss the conductance of the **TP1** breadboard.

A key feature of a molecular breadboard is that molecules can thermally populate multiple conformational states with distinct geometries and molecular conjugation, thereby dynamically creating new circuits with distinct conductance features. Thus, the molecular conformational space needs to be sampled for thermally accessible conformations. We therefore considered multiple optimized geometries of the **TP1** molecule obtained from different starting conformations which differed in the relative orientations of the aromatic rings (see Methods and section). The 18 optimized **TP1** geometries thus obtained were separated energetically by ∼3 kcal mol^–1^ (Fig. S5A in ESI†) and are accessible at room temperature. The optimized geometries of **TP1** differ (Fig. S5A†) in the relative positions of nitrogen atoms in the two terpyridine subunits and the torsion angles between the pyridine rings and between the pyridine and benzene rings. The largest variation in nitrogen atom pair distances (∼2.5 Å) was seen for atoms located in different terpyridine units (inset of Fig. S5A†). Although we constructed the initial geometries of **TP1** to have both, completely planar, and completely orthogonal orientations of adjacent terpyridine rings (see Methods and Section S.3 of ESI†), the optimized geometries showed a narrow range of torsion angles between adjacent pyridine rings of 25–30 degrees (Fig. S5B†) and pyridine benzene ring of 36–40 degrees (Fig. S5C†). Overall, all optimized structures exhibit similar π-conjugation with negligible differences in the HOMO–LUMO gap. To summarize, the energy landscape for the **TP1** molecule shows multiple thermally accessible conformations which would contribute to the conductance in MCBJ experiments. However, preliminary evaluation of the electronic structure and geometric parameters suggests that the conformational heterogeneity would likely to have a modest effect on the molecular conductance of the **TP1** molecular breadboard in break junction measurements.

### Conductance measurements on the **TP1** molecular breadboard

**Fig. 3:**
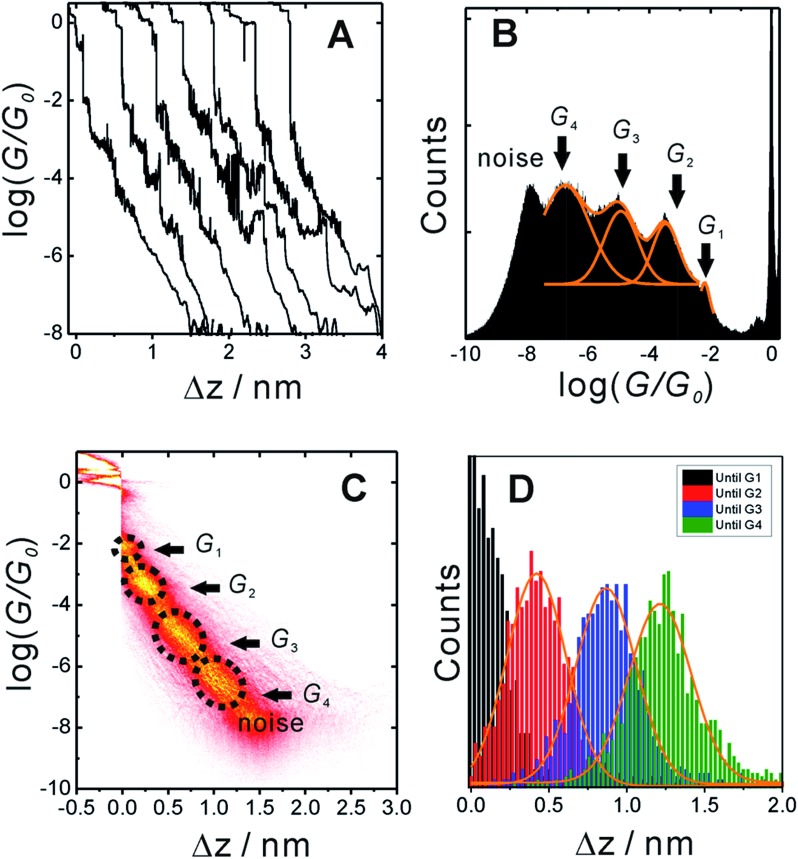
(A) Typical individual conductance *versus* relative displacement distance traces of **TP1** recorded in MCBJ experiments in 1,3,5-trimethyl benzene (TMB)/tetrahydrofuran (THF) (4 : 1, v/v) containing 0.1 mM of the target molecules. (B) 1D logarithmic conductance histograms constructed from more than 1000 individual curves and recorded with a bias voltage of 0.10 V. (C) 2D conductance histogram. (D) Characteristic length 1D histograms analysed for *G*_1_, *G*_2_, *G*_3_ and *G*_4_.

In order to measure conductance through all possible circuits of **TP1** molecular breadboard, we employed a MCBJ set-up capable of operating in solution.^9,11,39^ Fig. 3A shows typical conductance-distance traces obtained in the presence of **TP1** as recorded in 1,3,5-trimethylbenzene (TMB)/tetrahydrofuran (THF) (4 : 1, v/v). The breaking of atomic Au–Au contacts appears as plateaus observed in the conductance-distance traces in the range 1 ≤ *G*/*G*_0_ ≤ 10, where *G*_0_ = 2*e* ^2^/h is the quantum of conductance. Additional plateaus appearing in the range 10^–1^ *G*_0_ ≥ *G*/*G*_0_ ≥ 10^–7.5^ *G*_0_. The noise limit of our MCBJ setup under the current experimental conditions is reached at 10^–8^ *G*_0_. Fig. 3B displays the one-dimensional (1D) histogram on a logarithmic conductance scale as constructed from more than 1000 individual traces of **TP1**. The corresponding two-dimensional (2D) histogram of **TP1** is shown in Fig. 3C. The individual conductance–distance traces were normalized to the common point Δ*z* = 0 at *G* = 0.7 *G*_0_, which is characterized by a sharp drop in conductance immediately after breaking the last Au–Au monatomic contact upon pulling the leads apart. The quantitative analysis of the 1D and 2D histograms (Fig. 3B and C) reveals the existence of four well-defined conductance features for **TP1** whereas no features were observed in experiments without **TP1** molecules in solution (see Section S.2 in ESI†).

The presence of four distinct conductance features in the histograms of **TP1** (*G*_1_, *G*_2_, *G*_3_ and *G*_4_ in Fig. 3) can be attributed to the sequential formation of different contact configurations between the molecule and the gold leads. Quantitative analysis of the 2D conductance (Fig. 3C) and characteristic length (Fig. 3D) histograms provided the following information: the high conductance state “*G*_1_” is observed as a conductance cloud in the range 10^–3^ *G*_0_ ≤ *G*_1_/*G*_0_ ≤ 10^–2^ *G*_0_ centered around the most probable value 
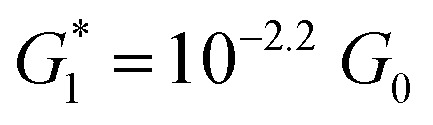
 at a relative lead displacement 
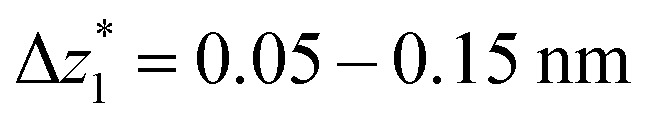
. Considering the snapback distance Δ*z*_corr_ = (0.5 ± 0.1 nm) upon breaking of the monatomic Au–Au contact,^9,11,53^ we estimate an absolute most probable gap distance 
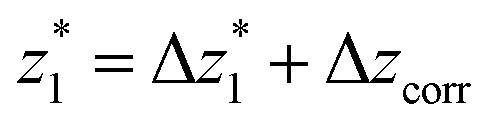
 in the range of 0.45 nm to 0.65 nm. The second highest conductance state “*G*_2_” is observed in the range 10^–4.5^ *G*_0_ ≤ *G*_2_/*G*_0_ ≤ 10^–3^ *G*_0_ with the most probable value 
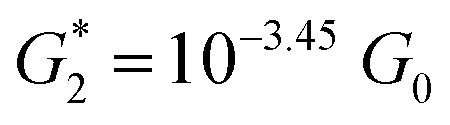
, and corresponding absolute gap distance 
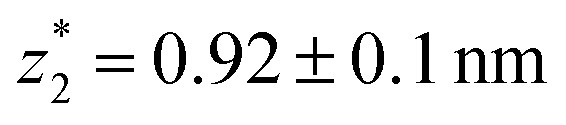
. Similarly, the next two conductance states *G*_3_ and *G*_4_ are seen in the range 10^–6.5^ *G*_0_ ≤ *G*_3_/*G*_0_ ≤ 10^–4.2^ *G*_0_ and 10^–7.8^ *G*_0_ ≤ *G*_4_/*G*_0_ ≤ 10^–6^ *G*_0_ and 
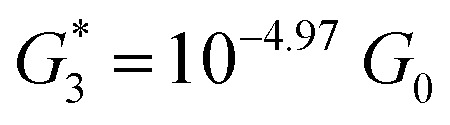
, 
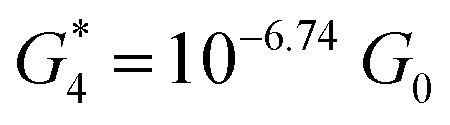
 respectively. The corresponding absolute most probable gaps for *G*_2_ and *G*_3_ conductance states are 
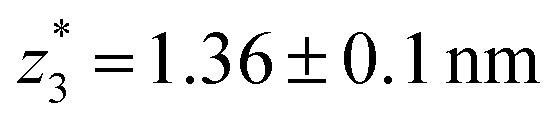
 and 
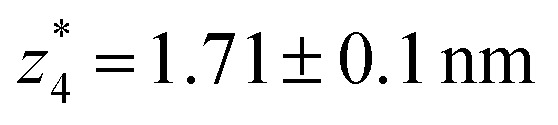
 respectively. These data clearly demonstrate that the single molecular junction conductance of **TP1** decreases in distinct steps upon pulling the gold leads apart and exhibits four conductance states.

### Conductance calculations for the **TP1** molecular breadboard

**Fig. 4:**
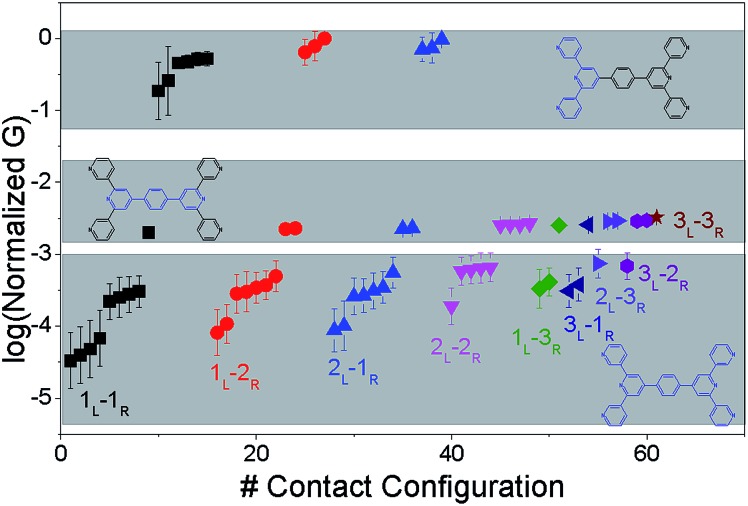
Conductance features of the **TP1** molecular breadboard circuits: average conductance (normalized with respect to the highest value) and variance over 18 optimized molecular conformations for the 61 circuits enumerated for **TP1**. The conductance data is coloured by the type of circuit as indicated and spread out across three bands. Fragments of **TP1** whose circuits contribute to each band are shown in blue colour. Error bars where not visible are smaller than the symbols.

The origin of the conductance features produced in the experiments (Fig. 3) should be examined in the context of the 61 possible circuits enumerated for the **TP1** molecular scaffold (*vide supra*) and their thermally accessible conformations. We thus carried out electronic structure calculations on the optimized **TP1** geometries and Green's function based tunneling transport calculations (see Methods) for each single and multi-terminal molecular circuit enumerated in Table 1 within the 18 optimized geometries (1098 circuit calculations). In Fig. 4 we show the average conductance and associated standard deviations for each of the 61 circuits enumerated in Table 1 calculated across the 18 optimized **TP1** geometries. The conductance of the single and multi-terminal circuits in the **TP1** breadboard spans almost five orders of conductance values which matches the span of the experimental conductance range (10^–2^ *G*_0_ to 10^–7^ *G*_0_) in Fig. 3.

Interestingly, the conductance values are distributed within 3 non-overlapping bands (gray color bands in Fig. 4), with the highest conductance band well separated from the remaining two. Each conductance band contains both single and multi-terminal circuits (colored by circuit type, *M*_L_–*N*_R_; *M*, *N* = 1, 2, 3) in Fig. 4. A striking feature of the conductance pattern in Fig. 4 is that the placement of circuits in each of the 3 conductance bands is independent of the number of contacts (single *vs.* multi-terminal) made by each electrode. In fact, multi-terminal circuit currents are only slightly higher (within a few fold) of corresponding single terminal currents within each conductance band. It can be shown (Section S.6 of ESI†) that for conditions (absence of correlated electronic injection, tunneling currents) assumed in this study, the currents from a multi-terminal circuit are simply a sum of single terminal currents. Thus, the three conductance bands in Fig. 4 can be primarily attributed to different single terminal circuits and their additive combinations (see Section S.6 of ESI†).

Each of the three conductance bands in Fig. 4 to correlate with the number of rings within contributing molecular circuits (see molecular fragments drawn in blue on the **TP1** scaffold in Fig. 4 for each band). Circuits within the **TP1** molecular breadboard can contain between 2–5 aromatic (purely pyridine, or a mix of pyridine and benzene) rings as shown in Table 1 and Fig. 2. The highest conductance band in Fig. 4 arises from contributions of 2-ring and 3-ring circuits within each terpyridine arm of **TP1** (*e.g.* L(S1)–R(S2) and L(S1)–R(S3) from Fig. 1B). The second conductance band arises from primarily from 3-ring circuits comprising of the core pyridine-phenyl-pyridine branch (L(S2)–R(S2′) from Fig. 1B). The lowest conductance band arises from 4-ring circuits which comprise pyridine-pyridine-phenyl-pyridine branches (*e.g.* (L(S1)–R(S2′) from Fig. 1B) and from 5-ring circuits (*e.g.* (L(S1)–R(S1′) or (L(S1)–R(S3′) from Fig. 1B) which span both the terpyridine arms and include the central phenyl ring.

The variation in conductance values across the different geometries is modest, less than an order of magnitude. In our optimization protocol, we generated distinct geometries sampling the widest possible range of dihedral angles for **TP1** (Section S.3 of ESI†). Nevertheless, the final optimized geometries showed a very narrow distribution of dihedral angles varying over a range of only ∼5 degrees (Fig. S5B and S5C†). Thus, this is the maximum variation in dihedral angles anticipated for thermally accessible **TP1** conformations and leads to an overall variation in computed conductance of less than one order of magnitude (Fig. 4). In contrast, the experimental conductance distributions (Fig. 3) are much broader and cannot be explained in terms of variation in **TP1** torsional flexibility alone. It is possible that the factors which are not included in our computational modeling, such as heterogeneity in the nitrogen–gold coordination geometries and the solvent effects may significantly contribute to broadening of the observed experimental conductance distribution.

Break junction experiments can access multiple circuits across a molecule with multiple electrode anchoring positions by changing the electrode separation.^17,18,38^ Experimental observables include conductance values and the most probable electrode separations at which they occur (Fig. 3C). Computationally we have access to the conductance of all 61 circuits (Fig. 4) of **TP1** and their terminal nitrogen atom separations (Fig. S5A in ESI†). To convert the information in Fig. 4 and S5A† to a conductance *vs.* electrode separation plot, we propose a simple intuitive model: any circuit for which the terminal N–N atom separation (averaged over the 18 optimized conformations; see inset of Fig. S5A in ESI†) spans the electrode gap will contribute to the conductance of **TP1** at that separation. For multi-terminal circuits the span of the shortest N–N separation distance is considered.

**Fig. 5:**
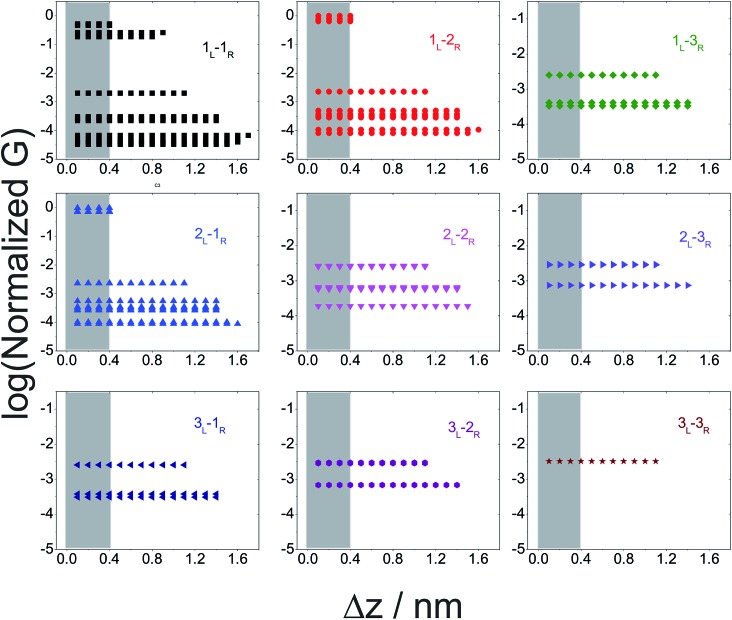
Contribution of single and multi-terminal circuits to **TP1** conductance as a function of electrode separation: in break junction experiments, different sets of circuits can be accessed by controlling the electrode separation. The different panels show average conductance (from 18 optimized geometries) contributions to the total conductance from different single and multi-terminal circuits (circuit notation and colours matching those in Fig. 4) whose terminal N–N distance (averaged over 18 optimized **TP1** geometries) span the junction gap as a function of electrode separation (Δ*z*). The shaded region indicates the conductance and electrode separations not accessible in MCBJ experiments as they lie below the estimated lower bound value of the snap-back distance (∼0.4 nm).

In Fig. 5 we plot the average conductance (calculated over 18 conformations) for all **TP1** circuits (from the set of 61) which spans the junction gap as a function of electrode separation. Contributions from the specific single and multi-terminal circuits considered in Fig. 4 are separated out into different panels (the colors of symbols in each panel match those adopted in Fig. 4). The shaded region in each panel of Fig. 5 represents electrode separations (and corresponding conductance values) which are inaccessible to MCBJ experiments as they fall below the minimum estimated value of the electrode snapback distance (snap back distance ≈ 0.4–0.6 nm). Fig. 5 shows that all 61 circuits of **TP1** are accessible to MCBJ experiments at short electrode separations (Δ*z* < 0.4 nm). As the electrode separation increases from 0.4 nm to the full molecular length of 1.6 nm, the number of circuits contributing to the conductance of **TP1** decreases as the terminal N–N atom separations for some circuits cannot span the junction gap. For instance, the highest conductance single terminal (1_L_–1_R_) circuit does not contribute to the **TP1** conductance at electrode separations of 0.5 nm and beyond. Thus, as circuits drop out, the total conductance is expected to reduce in discrete steps as a function of electrode separation. The MCBJ experiment measures only the total conductance as a function of electrode separation (Fig. 3C) which can be obtained by summing up the conductance values from all circuits across the different panels in Fig. 5 at each electrode separation.

## Discussion

Our results demonstrate the potential of state-of-the-art break junction experiments in accessing multiple circuits within a single molecular scaffold. While the **TP1** molecule contains a large number (61) of possible circuits, there are degeneracies in many conductance values (see Fig. 4) as well as the terminal distances (see inset of Fig. S5A in ESI†) for the circuits. Further, multiple circuits may contribute at a given electrode separation, reducing the number of distinct features captured in experimental conductance *vs.* electrode separation traces (4 peaks observed for **TP1**). In order to develop the concept of a molecular breadboard further, it is critical to map the experimental conductance features to the underlying contributing circuits. Note that a molecular breadboard is expected to have features distinct from a macroscopic electrical breadboard. First, molecules such as **TP1** exhibit thermally driven conformational flexibility which can vary the conductance of individual circuits by an order of magnitude (Fig. 4) or more.^10,54^ Second, quantum interference effects can produce non-trivial additive effects of currents from multiple channels^55,56^ and make currents sensitive to the anchoring group positions on aromatic rings.^14,31,57,58^ In the following sections, we discuss circuit assignments for the experimentally observed conductance features and the effect of quantum interference and thermal fluctuations on the conductance of **TP1**. Finally, we also discuss the requirements for formulating circuit rules of molecular breadboard circuits. The analytical framework as well as key ideas developed here should be useful to interpret the data from experiments which examine multiple channels of charge flow within a single molecular scaffold.^38^

### Assignments of circuits to the experimentally observed conductance states

**Fig. 6:**
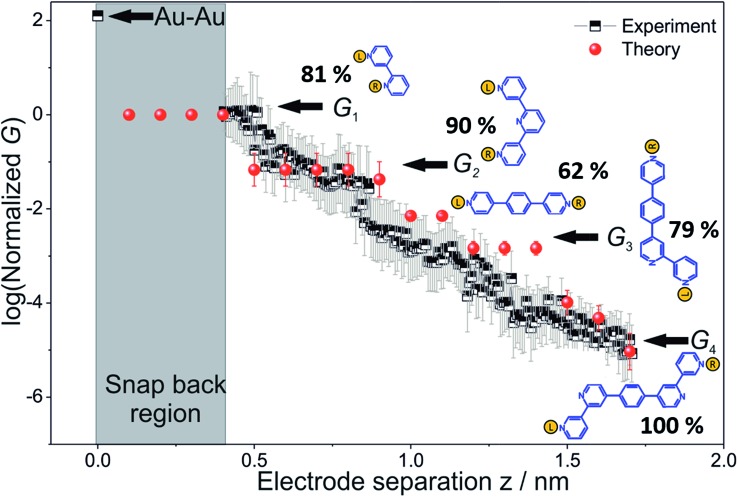
Conductance map of **TP1** molecular breadboard as function of electrode separation: comparison 2-D plots of computed total conductance from 61 circuits (red symbols) and master curve extracted from 2D-histogram of MCBJ measurements (black and white symbols). The 2D master curve was constructed by estimating the most probable conductance values from the Gaussian fits to cross sections of 2D histogram at different displacement positions Δ*z* in Fig. 3c. The contributions of each of the 61 circuits to the computed total conductance were averaged over 18 optimized **TP1** geometries. Since our computations only capture relative trends, both computed and experimental conductance values were normalized with respect to the conductance values at the smallest electrode separation (*G*_1_). Error bars for the computed conductance, where not visible, are smaller than the symbols. The dominant circuits (gold spheres indicate left/right electrode contacts) contributing to each conductance state are shown along with estimates of their percentage contribution to the total conductance at specific electrode separations where conductance steps occur. The shaded region indicates the conductance and electrode separations not accessible in MCBJ experiments as they lie below the estimated lower bound value of the snap-back distance (∼0.4 nm).

To derive the computed conductance *vs.* electrode separation plot shown in Fig. 5, we assumed a model wherein all circuits with terminal N–N distances equal to or larger than the junction gap contribute to the total **TP1** conductance at that separation. Physically, since the molecule spans the gap, nitrogen atoms can coordinate with gold atoms located at the tip or further away from the gap (see Fig. 2 and S6A†). Using this model, Fig. 5 shows that for an electrode snap-back distance of Δ*z*_corr_ = 0.4–0.6 nm (see Section S.2 in ESI† for an experimental determination),^9,11,12^ all 61 circuits contribute to the conductance and these circuit currents can be summed up to obtain the junction conductance at 0.4–0.6 nm electrode separation. As the electrode separation increases, circuits with terminal N–N distances shorter than the electrode separation drop out and the conductance is expected to reduce, thereby producing steps in conductance. For tunneling transmission, the relative contribution of various circuits are expected to be exponentially weighted by their terminal N–N distances. However, quantum interference and thermal fluctuations can significantly modulate the relative contributions of various circuits (*vide infra*). In Fig. 6 we plot the total junction conductance at each electrode separation (sum of conductance values from all contributing circuits across the 9 panels in Fig. 5) along with the experimental data (from Fig. 3C). Both computed and experimental data are normalized with respect to their corresponding highest conductance values in Fig. 6. The computed conductance is in excellent agreement with experimental data in terms of both, the overall range, and the drop of the conductance values with electrode separation validating our model. The computational model captures all four experimentally observed conductance states *G*_1_, *G*_2_, *G*_3_, and *G*_4_ and their correlation with electrode separation. The computational results suggest that there may be a fine structure to the *G*_3_ conductance profile near 1 nm which may not be resolved in the experiments. Further, the computational analysis indicates that the MCBJ experiments contact all 61 circuits and probe the full range of conductance values of the **TP1** breadboard circuit within the experimentally accessible electrode separations (0.4–1.7 nm). In Fig. 6 we also show the dominant circuits contributing to each conductance state along with estimates of their percentage contribution. In the next paragraph, we further discuss how these circuits were assigned and their contributions estimated for each conductance state.

**Fig. 7:**
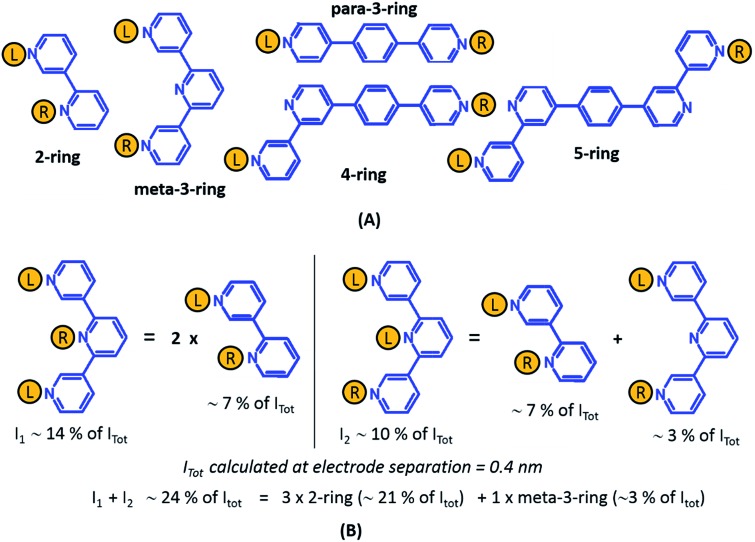
Decomposition of multi-terminal circuits into constituent single terminal circuits. (A) Five classes of single terminal circuits (gold spheres indicate left/right electrode contacts) present in the **TP1** molecular breadboard. (B) Two of the highest contributing circuits (current *I*_1_ ∼ 14% and current *I*_2_ ∼ 10% contribution) to the total current *I*_tot_ at electrode separation of 0.4 nm (Fig. 5 or 6) and their single circuit decomposition. *I*_1_ is a sum of currents from two 2-ring circuits. *I*_2_ is a sum of currents from one 2-ring circuit and one *meta*-3-ring circuit. *I*_tot_ at electrode separation of 0.4 nm is obtained by summing up contributions from 61 such circuits (see Fig. 4B). In the example the current *I*_1_ + *I*_2_ amounting to 24% of *I*_tot_ is a sum of currents from three 2-ring circuit (∼21% of *I*_tot_) and one *meta*-3-ring circuit (∼3% of *I*_tot_).

From the data in Fig. 5 we find that a total of 61, 51, 49–53, and 5–9 single and multi-terminal circuits contribute to the computational *G*_1_, *G*_2_, *G*_3_, and *G*_4_ conductance states respectively. Note that we obtain a range of circuit combinations for the *G*_3_ and *G*_4_ conductance states as these states show a much stronger drop in conductance with electrode separation than the *G*_1_, and *G*_2_ conductance states (see also Fig. 6). Circuits can then be assigned to each conductance state based on the following two observations: (1) for the deep tunneling charge transport regime operational in **TP1**, the current from all multi-terminal circuit contributions can be decomposed into the sum of currents from all constituent single-terminal circuits, and (2) there are only 5 types of single terminal circuits (see Fig. 7A) present in **TP1**. These two conditions together imply that the total conductance of **TP1** at each electrode separation can be expressed as a linear combination of currents (or conductance values) from the 5 single terminal circuits shown in Fig. 7A. In Fig. 7B, we show examples of the procedure to decompose two multi-terminal circuits with significant contributions to the total conductance of **TP1** at an electrode separation of 0.4 nm into single terminal 2-ring and *meta*-3-ring circuits. We applied the decomposition procedure shown in Fig. 7B to express the current (conductance) from all contributing circuits in terms of the 5 types of single terminal circuits shown in Fig. 7A and estimated their relative contributions to the total conductance at each electrode separation. Further, the decomposition procedure also provides us with the total number of single terminal circuits contributing to a given conductance state. The circuits with the dominant contributions are shown in Fig. 6 and the detailed circuit assignments for the four conductance plateaus (*G*_1_, *G*_2_, *G*_3_, and *G*_4_) are presented in Table 2. Finally, coupling the information in Table 2 with the experimentally measured values for each conductance state and the percentage contribution to these states from the 2–5 ring circuits, absolute conductance values for each single terminal circuits assigned in Fig. 5 can be extracted. For instance, using the data in Table 2 and the measured *G*_1_ conductance, we can estimate the 2-ring circuit conductance to be: *G*(2-ring) = (0.81 × *G*_1_(experiment))/12. On the other hand the 3-ring circuit conductance can be estimated in two ways, (1) either using the experimental *G*_1_ value: (*meta*-3-ring) = (0.18 × *G*_1_(experiment))/6, or using the measured *G*_2_ conductance: *G*(*meta*-3-ring) = (0.90 × *G*_2_(experiment))/2. Estimates of absolute conductance values for all 5 single terminal circuits (2-ring, *meta*-3-ring, *para*-3-ring, 4-ring, and 5-ring) obtained by this procedure are listed in the last row of Table 2 along with the experimental conductance values from which they are drawn. Estimates for single terminal circuits calculated from multiple experimental conductance states were found to be consistent (differing by less than two fold in Table 2).

**Table 2:** Single terminal (2–5 ring) circuit contributions in terms of number of circuits and percentage contributions to the total conductance for the four different conductance states observed in experiments. The electrode separations at which the circuit contributions were mapped for each conductance state are indicated in brackets in the first column. The last row indicates absolute single terminal currents estimated from the experimentally measured conductance values which are shown in brackets. Since the contributions of single terminal circuits overlap for different conductance states, it is possible to estimate the conductance of some single terminal circuit in two ways. For such cases both estimates are shown along with the experimental conductance states from which they are drawn

Conductance state	Contribution of single terminal (2–5 ring) circuits to each conductance state: number of circuits (percentage contribution to total conductance)				
	2-Ring	*meta*-3-Ring	*para*-3-Ring	4-Ring	5-Ring
*G*_1_ (0.4–0.5 nm)	12 circuits (81%)	6 circuits (18%)	16 circuits (<1%)	64 circuits (<1%)	64 circuits (<1%)
*G*_2_ (0.5–0.9 nm)	0	2 circuits (90%)	16 circuits (6%)	64 circuits (3%)	64 circuits (<1%)
*G*_3_ (1.0–1.1 nm)	0	0	16 circuits (62%)	64 circuits (32%)	64 circuits (6%)
*G*_3_ (1.2–1.4 nm)	0	0	0	32 circuits (79%)	48 circuits (21%)
*G*_4_ (1.6 nm)	0	0	0	0	7 circuits (100%)
*G* (single-terminal) estimates	(*G*_1_) 4.25 × 10^–4^ *G*_0_	(*G*_1_) 1.89 × 10^–4^ *G*_0_	(*G*_2_) 1.35 × 10^–6^ *G*_0_	(*G*_2_) 2.16 × 10^–7^ *G*_0_	(*G*_3_ – 1.2 nm) 4.81 × 10^–8^ *G*_0_
		(*G*_2_) 1.62 × 10^–4^ *G*_0_		(*G*_3_ – 1.2 nm) 2.72 × 10^–7^ *G*_0_	(*G*_4_) 2.57 × 10^–8^ *G*_0_

The conductance plateaus in Fig. 3 and 6 drop exponentially with distance. By plotting the most probable conductance values extracted from the 1D conductance histogram (Fig. 3B) as a function of electrode separations extracted from characteristic length histogram (Fig. 3D), we estimated an experimental tunneling decay constant as *β*_Experiment_ = 3.4 nm^–1^ (Section S.9 of ESI†). Since the theoretical analysis in this section assigns specific dominant 2–5 ring single terminal circuits to each conductance plateau in Fig. 6, we also plotted the theoretically calculated conductance of the dominant circuits as a function of electrode separation (Fig. S11B†). The conductance from dominant circuits drops exponentially with increase in the number of rings of the circuits with a decay constant of *β*_Theory_ = 3.8 nm^–1^ (Section S.9 of ESI†). The decay constant values extracted from both the experiment and theory are in similar range with the decay constant values reported previously for oligophenyls (OP: 3.5–5 nm^–1^), OPE (2.0–3.4 nm^–1^), and oligophenyleimine (OPI: 3 nm^–1^).^6^ However, we stress that the interpretations of tunneling decay constants for circuits embedded within a molecular scaffold is different and more complex than that for isolated circuits. For instance, the drop in **TP1** junction conductance occurs not only due to a change in the dominant circuit (increasing number of rings) with electrode separation, but also due to a drop in the number of contributing circuits (see Fig. 5). Further, the dominant circuits within **TP1**: (1) are composed of mixture of pyridine and phenyl rings, (2) do not have linear ring connectivity, and (3) differ in their ring connectivity positions (*ortho*/*meta*/*para*) to the electrodes. The next section sheds further light on the relative conductance values of the dominant circuits assigned in Fig. 6 and how they shape the observed conductance features of **TP1**.

### Quantum interference effects and their manifestations in the conductance features of **TP1**

Several studies have reported quantum interference effects in molecular junctions.^14,31,41,57,58^ Xing *et al.* found that phase cancellation of currents through molecular orbitals of opposite symmetry diminished the contribution of frontier orbitals to both electron and hole transport in OPE molecules.^41^ Arroyo *et al.* found interference effects between charge transport pathways through HOMO and LUMO of a benzene ring coupled to thienyl anchoring units through ethynyl spacers.^57^ Further, anchoring group placement at *ortho*-, *meta*-, or *para*-positions of six membered rings have been shown to significantly modulate the conductance of conjugated systems.^14,58^

**Fig. 8:**
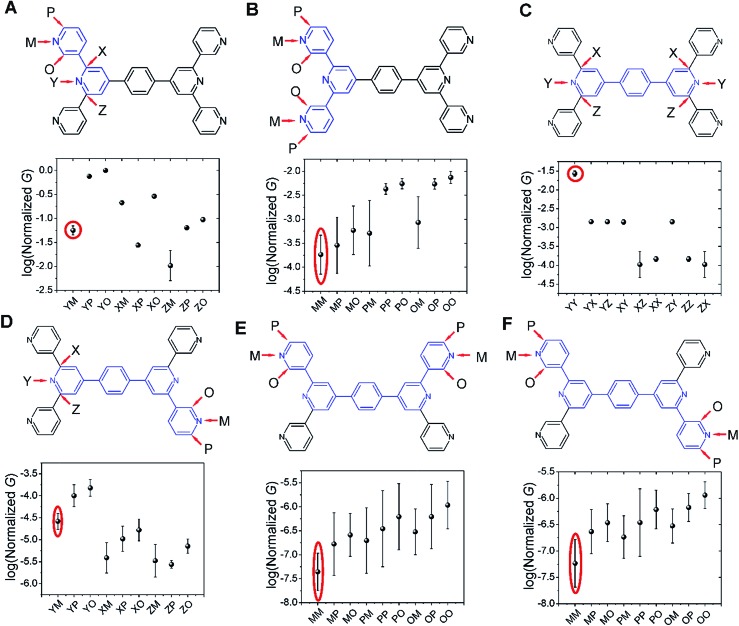
Quantum interference effects in **TP1** molecular breadboard: the six panels (A–F) show single terminal conductance values (average and standard deviation from 18 different optimized geometries for each circuit) for 2–5 ring embedded fragments within **TP1**. All conductance values were normalized with respect to the highest conductance for the 2-ring circuit in panel (A). The 2–5 ring circuits corresponding to each panel are highlighted in blue over the **TP1** scaffold. The positions of atoms electronically coupled to the left and right electrodes are labelled as *para* (*P*), *meta* (*M*), and *ortho* (*O*) with respect to the connectivity to the adjacent ring. Since the central ring of each terpyridine arm is connected to two terminal pyridine rings and also to the central benzene ring, the electrode contacts for the central pyridine ring of each terpyridine unit are labelled as X, Y and Z. For a 2-ring circuit (panel A) Y = *ortho*, Z = *meta* and for 3-ring/4-ring circuits X = Z = *meta* and Y = *para*. In our calculations all molecule-electrode electronic couplings have the same value (*Γ* = 0.1 eV). The data circled in red are from dominant 2–5 circuits assigned for the **TP1** conductance states (Fig. 6). Note that the normalization for all panels is with respect to the highest conductance values in panel (A), which are for carbon terminated circuits which are not considered in the calculations in Fig. 4–6. Further, the electrode accessibility of all nitrogen atoms is not the same for **TP1** (see eqn (1) in Methods). Thus the range of conductance values is larger than that for the data in Fig. 4–6.

The dominant 2–5 ring circuits assigned to the **TP1** junction conductance at different electrode separations show diverse terminal nitrogen placements, and in this section we examine interference effects in these circuits. For each 2–5 ring embedded fragment of **TP1**, we computed conductance for electrode electronic couplings with atoms at *para*-, *meta*-, and *ortho*-positions of the pyridine rings of the molecule (see markings for each circuit in Fig. 8). Note that all 2–5 circuit conductance computations (Methods) use the full **TP1** Hamiltonian (*i.e.* calculations are on embedded circuits). In order to cover the three *para*-, *meta*-, and *ortho*-connectivities for each 2–5 ring fragment, we considered carbon atom terminated circuits in addition to the dominant circuits assigned in the last section. While, previous studies have suggested that the electrode can couple to the carbon backbone through the π orbitals.^6,8,14,59–61^ It is not our intent to explore the through space coupling effect here. Rather, the carbon terminated circuits serve as surrogates of nitrogen terminated circuits to assess the effect of ring-electrode connectivity on the conductance of each 2–5 ring embedded fragment of **TP1**. In Fig. 8 we plot average values and standard deviation for single terminal circuit conductance values sampled across the 18 different optimized geometries of **TP1**.

Following only the conductance values of the dominant 2–5 ring systems (red circles) across the panels, we find *G*_avg_(2-ring) ∼ *G*_avg_(*para*-3-ring) ≫ *G*_avg_(*meta*-3-ring) > *G*_avg_(4-ring) ≫ *G*_avg_(5-ring). Clearly, the conductance drop with electrode separation does not correlate with the increase in the tunneling length (number of aromatic rings) of the dominant circuits contributing to the conductance at increasing electrode separations. The data in Fig. 8A and C show that quantum interference effects can lead to significant overlaps in the conductance ranges for molecular circuits of different length: the optimally placed *para* anchors produce the highest possible *para*-3-ring conductance (Fig. 8C), while the sub-optimally placed *meta* and *ortho* anchors produce a weak 2-ring conductance (Fig. 8A). Thus, if all nitrogen atoms had the same accessibility to the electrodes, the **TP1** breadboard would produce nearly degenerate *G*_1_ and *G*_2_ conductance values at the first two electrode separations (see for example Fig. S9†). Thus, quantum interference can produce tunneling length independent conductance features in the **TP1** breadboard. However, these signatures of quantum interference do not manifest in the experimental conductance *vs.* distance plots (Fig. 3C or 6). The nitrogen atoms of the central pyridine ring of the terpyridine units (core nitrogen atoms) are not as accessible to the electrode as nitrogen atoms of the peripheral pyridine rings. Based on a structural analysis on the optimized geometries of **TP1**, we estimate (see Discussion following eqn (1) in Methods) an effective ∼100 fold reduction in the electronic coupling for the core nitrogen atom relative to the peripheral nitrogen atoms of each terpyridine ring. In this case, the *G*_2_ conductance primarily arising from the 3-ring circuit (core nitrogen atoms at both terminals) in Fig. 7C is lowered relative to the *G*_1_ conductance primarily arising from the 2-ring circuit (only one terminal core nitrogen) as observed in the experiments (Fig. 3C). We present plots of the transmission for the dominant 2–5 ring circuits in Fig. S12 of ESI.† The plots reveal weak interference features due to the effect of conformational fluctuations for *meta*-3-ring circuits. Other circuits also show weak interference features as they are do not have *meta* terminated contacts at both ends.

In Fig. 8, we find that the lowest currents generally come from circuits which involve at least one *meta*-anchoring group position and the highest currents involve an *ortho*-/*para*-anchor. For instance, in 2-ring circuits (Fig. 8A), the average conductance: *G*_avg_(YP/YO) ≫ *G*_avg_(ZM). In the 3-ring circuits of Fig. 8B and C, we find *G*_avg_(PP) ≫ *G*_avg_(MM) and *G*_avg_(YY) ≫ *G*_avg_(XX/ZZ/XZ) respectively. We find that the effect of the anchoring group ring position on circuit conductance is preserved even when the terminal electrode contacting pyridine rings are separated by intervening aromatic rings. For instance, in 4-ring circuits (Fig. 8D), we find *G*_avg_(YP) ≫ *G*_avg_(XM/ZM) and in 5-ring circuits (Fig. 8E and F) we find *G*_avg_(PP) ≫ *G*_avg_(MM). In accordance with previous reports,^14^ we find higher currents for *para*-3-ring circuits relative to that for *meta*-3-ring systems (*G*_avg_(PP) in Fig. 8B ≪ *G*_avg_(YY) in Fig. 8C). Other trends get washed out due to variations in conductance which arise from the torsional flexibility of **TP1**. The largest variations in conductance are seen for 5-ring circuits (Fig. 8E and F) which wash out the conductance differences for *para*-, *meta*-, and *ortho*-terminated circuits. In contrast, conductance data for the 2- and *para*-3-ring circuits in Fig. 8A and C show much smaller fluctuations with well resolved conductance features. While 4-ring circuits (Fig. 8D) also show significant conductance fluctuations, two classes of conductance values are resolved; the YP (*para*–*para*) and YO (*para*–*ortho*) circuits show higher conductance values relative to the other circuits. The *meta*-3-ring terpyridine circuit in Fig. 8B also exhibits two cleanly resolved classes of circuits with low conductance and large fluctuations for at least one *meta*-anchor (MM, MP, MO, PM, and OM) and high conductance with low fluctuations for *ortho*-/*para*-anchors (PP, PO, OP, and OO). To summarize, we find that after including the effects of conformational heterogeneity, the modulation of conductance due to quantum interference is cleanly resolved for 2-ring and *para*-3-ring circuits (Fig. 8A and C), partially resolved for the *meta*-3-ring and 4-ring circuits (Fig. 8B and D), and washed out for the 5-ring circuits (Fig. 8E and F).

The results in this section show that the **TP1** molecular breadboard offers several circuits with coherent quantum conductance features. However, physical considerations such as the variable accessibility of the anchoring groups to the electrode and conductance fluctuations from the torsional flexibility of the molecule mask the signatures of quantum interference leading to the observed conductance features in Fig. 6. We also note that other factors not explored here, such as through space electronic coupling of electrode to the carbon backbone through the π orbitals,^6,8,14,59–61^ could also limit the modulation of observed conductance through quantum interference effects.

### Towards formulation of circuit rules for molecular breadboard circuits

Predicting the conductance through a molecular circuit in terms of transport properties of individual molecular components is an unsolved fundamental challenge in field of molecular electronics. From elastic scattering theory, transport through a molecular super circuit formed by binding two oligomers with transparencies T1 and T2 in series is proportional to T1 × T2.^55,62,63^ In accordance with this rule, several molecular wires show additive decay constants with increase in molecular length.^9,25^ Under the series rule above, the conductance of 4-ring and 5-ring circuits in **TP1** (Fig. 7A) should be a product of 2-ring and *para*-3-ring circuit conductance: *G*(4-ring) = *G*(2-ring) × *G*(*para*-3-ring) and *G*(5-ring) = *G*(2-ring) × *G*(*para*-3-ring) × *G*(2-ring). The data in Table 2 shows that the simple series rule does not apply for breadboard circuits; the estimated 4-ring and 5-ring conductance values are orders of magnitude larger than that anticipated by the series product rule describe above.

Joachim and co-workers have theoretically examined superposition rules in the context of molecular junctions where multiple molecular units were connected either in series or parallel to form super-circuits.^55,62^ Venkataraman and co-workers,^64^ verified the parallel superposition law proposed by Joachim and co-workers,^55^ and found evidence for constructive quantum interference in parallel superposition of molecular units in single molecular junctions. For **TP1**, single terminal circuits form channels which combine to yield each of the four conductance states observed for the breadboard. Thus, it is reasonable to ask if the conductance of the breadboard can be predicted from conductance data on isolated single terminal subcircuits. To this end, we measured the conductance of two single terminal circuits in isolation (see ESI:† Section S.2): molecules **R1**, and **R2** equivalent to the 2-ring circuit, and central core *para*-3-ring circuits in Fig. 7A. 81% of the *G*_1_ conductance for the **TP1** breadboard is assigned to the 2-ring circuit (Fig. 6). The absolute value of 2-ring conductance calculated in Table 2 (4.3 × 10^–4^ *G*_0_) appears to be in good agreement with the experimentally measured high conductance of the **R1** molecule (*G* **_R1_** ^H^ = 6.8 × 10^–4^ *G*_0_). However, this apparent match is misleading since the **R1** molecule and the 2-ring circuit within **TP1** differ in two crucial ways: (1) the accessibility of the terminal nitrogen atoms to electrodes in the **TP1** 2-ring circuit is not the same as in **R1**, and (2) the electronic structure of the **TP1** 2-ring system is coupled to that of the **TP1** framework, whereas **R1** represents an isolated 2-ring system. Contrary to the conventional macroscopic breadboard circuits, where adding one extra branch to the circuit does not influence the electrical properties of the other subcircuits, any new branch added to a molecule effectively creates a new molecule with different electronic properties.^55,62^ The differences between the component single terminal circuits in isolation and within the **TP1** breadboard is more evident for the *para*-3-ring circuit wherein the estimated 3-ring circuit conductance in Table 2 (1.35 × 10^–6^ *G*_0_) is two orders of magnitude lower than the experimentally measured conductance for the **R2** molecule (1.35 × 10^–4^ *G*_0_). Further, isolated subcircuits may have distinct conductance features which may not show up in the conductance features of their host breadboard. For instance, the **R1** circuit has a low conductance feature (*G* **_R1_** ^L^ = 1.3 × 10^–5^) which should not show up in the **TP1** breadboard as the most probable junction gap for this configuration (0.9 nm) indicates a dimer configuration (the terminal N–N distance for a 2-ring system is ∼0.4–0.5 nm).

The observations in this subsection indicate that more studies on differences, in terms of both geometry and electronic structure, of subcircuits in isolation and within larger molecular scaffolds such as **TP1** are necessary to build a reliable circuit theory for breadboard circuits.

## Conclusions

We have experimentally demonstrated for the first time four distinct conductance steps separated over 5 orders of magnitude (10 ^–2^ *G*_0_ to 10 ^–7^ *G*_0_) within a single molecular scaffold (Fig. 3). We modelled the conductance using a general theoretical framework which accounts for all possible circuits within the breadboard as well as the conformational flexibility of **TP1**. The multi-anchoring configurations in the **TP1** molecule were shown to create 61 single and multi-terminal circuits with conductance features spanning 5 orders of magnitude (Fig. 4). Molecular torsional flexibility created relatively modest modulations (about one order of magnitude) in the conductance (Fig. 4). The conductance steps for the **TP1** breadboard in the MCBJ experiments originate from distinct combinations of the 61 circuits at different electrode separations (Fig. 5). Based on our analysis we were able to assign specific 2–5 ring circuits to the experimentally observed conductance peaks (Fig. 6). We determined estimates of percentage contribution to total conductance at each electrode separation and absolute conductance values for the single terminal circuits (Fig. 6 and Table 2). Effects of quantum interference on the conductance features were examined and found to create degenerate conductance values in 2-ring and 3-ring circuits (Fig. 8). However, physical considerations based on the accessibility of the pyridyl anchoring groups to the electrode and thermal fluctuations were found to suppress the effects of quantum interference to produce the observed modulations in conductance which span 5 orders of variation in break junction experiments (Fig. 8). We have shown that the NEGF based theoretical framework can provide robust analysis of relative conductance values in breadboard circuits such as **TP1**. The combination of theory and experiment presented in this paper provides guidelines for rational molecular design enabling the access to specific circuits and conductance features in future experiments. In this context, development of circuit theory for molecular breadboards through a systematic study of component circuits was discussed.
