# Supplementary material for: Conductance in a bis-terpyridine based single molecular breadboard circuit
Source: Chem Sci. 2016 Nov 3;8(2):1576–91. doi: 10.1039/c6sc03204d (PMC5359913; doi:10.1039/c6sc03204d)
Supplement: Supplementary file 1 [file SC-008-C6SC03204D-s001.pdf]

Electronic Supplementary Material (ESI) for Chemical Science.
This journal is © The Royal Society of Chemistry 2016

Supplementary Information

Conductance in a Bis-Terpyridine Based Single Molecular Breadboard Circuit

Charu Seth[†#], *Veerabhadrarao Kaliginedi*[‡#]*, *Sankarrao Suravarapu,* [‡]*David Reber,* [‡] *Wenjing Hong,* [Θ] *Thomas Wandlowski*[‡], *Frédéric Lafolet,* [⊥] *Peter Broekmann,* [‡] *Guy Royal,* [⊥]* *Ravindra Venkatramani*†*

†Department of Chemical Sciences, Tata Institute of Fundamental Research, Homi Bhabha Road, Colaba, Mumbai 400 005, India.

‡Department of Chemistry and Biochemistry, University of Bern, Freiestrasse 3, CH-3012, Bern, Switzerland.

Θ Department of Chemical and Biochemical Engineering, College of Chemistry and Chemical Engineering, Xiamen University, Xiamen 361005 (China).

⊥ Université Grenoble Alpes, Département de Chimie Moléculaire, UMR CNRS-5250, Institut de Chimie Moléculaire de Grenoble, FR CNRS-2607, BP 53, 38041 Grenoble Cedex 9, France.

# These authors contributed equally

* Corresponding Authors

Veerabhadrarao Kaliginedi, Email: veera.kaliginedi@epfl.ch

Guy Royal, Email: guy.royal@univ-grenoble-alpes.fr

Ravindra Venkatramani, Email: ravi.venkatramani@tifr.res.in

S.1 Synthesis of TPI and associated molecules

All purchased chemicals and solvents were used as received.

Synthesis of 4′,4′′′′-(1,4-Phenylene)bis(3,2′:6′,3′′-terpyridine) (TP1). TP1

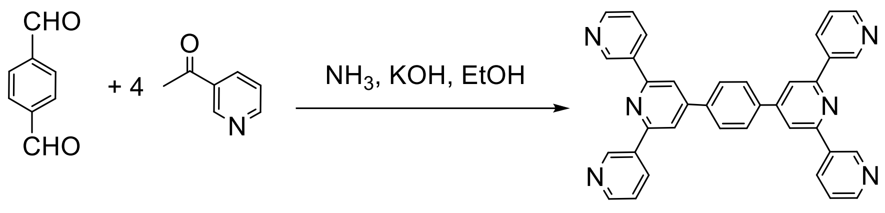

TP1

TP1 was prepared as recently reported by Yoshida et al., [1]using the one-pot synthesis previously developed by Hanan et al.[2] Spectral data are in accordance with the literature report.[2]

Synthesis of 1,4-bis(4-pyridinyl)benzene (R2)

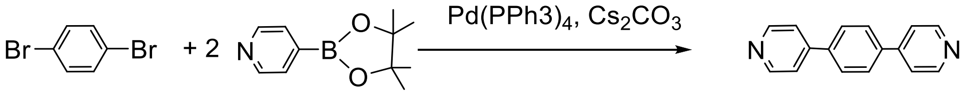

1,4-bis(4-pyridinyl)benzene was prepared from 1,4-dibromobenzene following the procedure reported by Su et al.[3] Spectral data are in accordance with the literature report.[3]

Synthesis of 2,3'-bipyridine (R1)

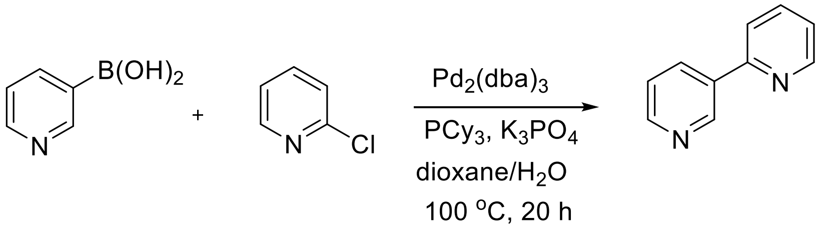

To a solution of pyridin-3-ylboronic acid (676 mg, 5.5 mmol), $Pd_2(dba)_3$ (46 mg, 0.05 mmol), and PCy_3 (34 mg, 0.12 mmol) in dioxane (13.35 ml), was added 2-chloropyridine (568 mg, 5 mmol), K_3PO_4 (1.80 g, 8.5 mmol) (solution in water 6.65 ml) successively under inert atmosphere. The resulting solution was stirred at 100 °C for 20 h. This reaction was carried out in a dry two neck round bottom flask. After the reaction mixture was filtered over a pad of silica gel and washed with ethyl acetate. The filtrate was concentrated under reduced pressure and the aqueous phase was extracted with ethyl acetate three times. The combined organic layers were dried over anhydrous sodium sulphate, concentrated under reduced pressure to get the crude

compound. Purification by silica gel column chromatography afforded orange oil. Spectral data is in accordance with the literature report [4].

S.2. MCBJ experiments and data analysis

Details of our MCBJ set up and data analysis procedures were introduced and discussed in our previous publications given as ref.[5-7] All the measurements were performed by using 0.1mM concentration of target molecule in solvent 1,3,5-trimethylbenzene(TMB) +Tetrahydrofuran (THF) (4:1 v/v ratio).

Calibration of MCBJ conductance –distance curves and Snap back distance correction (Δz_{corr}): The conductance-distance curves recorded by the MCBJ set-up were calibrated with

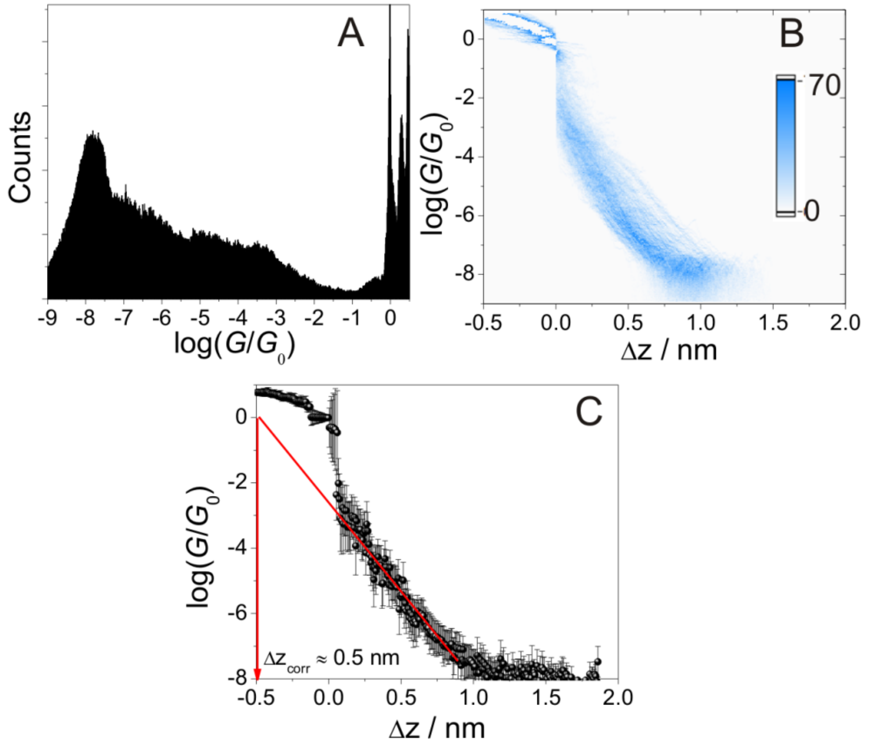

Figure S1: (A) 1D conductance histogram of solvent only measurement recorded at 100 mV bias. (B) 2D histogram generated from approximately 500 individual curves. (C) Master trace calculated from the 2D histogram. Master curves were constructed by calculating the most probable conductance values and standard deviation from the Gaussian fits to cross sections of 2D histogram at different displacement positions Δz.

using an assumption that the tunnelling decay is identical to that in a STM-BJ setup under the same experimental conditions (in presence of only solvent 1,3,5-trimethylbenzene(TMB)

+Tetrahydrofuran (THF) (4:1 v/v ratio) and decay constant observed in STM-BJ setup is ($\log(\Delta G/G_0)/\Delta z = 5.5\text{-}6\ nm^{-1}$).[7]

In a break junction experiment, immediately after breaking a gold-gold contact, the conductance of the junction drops to approximately $10^{-3}\ G_0$. Due to the so-called "snap-back" effect the gap between the two gold electrodes increases instantaneously to a certain distance Δz_{corr}. The snap-back distance is estimated, based on the analysis of the tunneling tail of conductance distance curves (recorded in presence solvent only), typically in the range between $10^{-3}\ G_0$ and $10^{-6}\ G_0$, which is estimated as $\Delta z_{corr} = 0.5 \pm 0.1$ nm (Supplementary Fig. S1).[5, 7, 8] To estimate the most probable absolute electrode separation ($z_i^* = \Delta z_i^* + \Delta z_{corr}$) we used snap back distance correction.

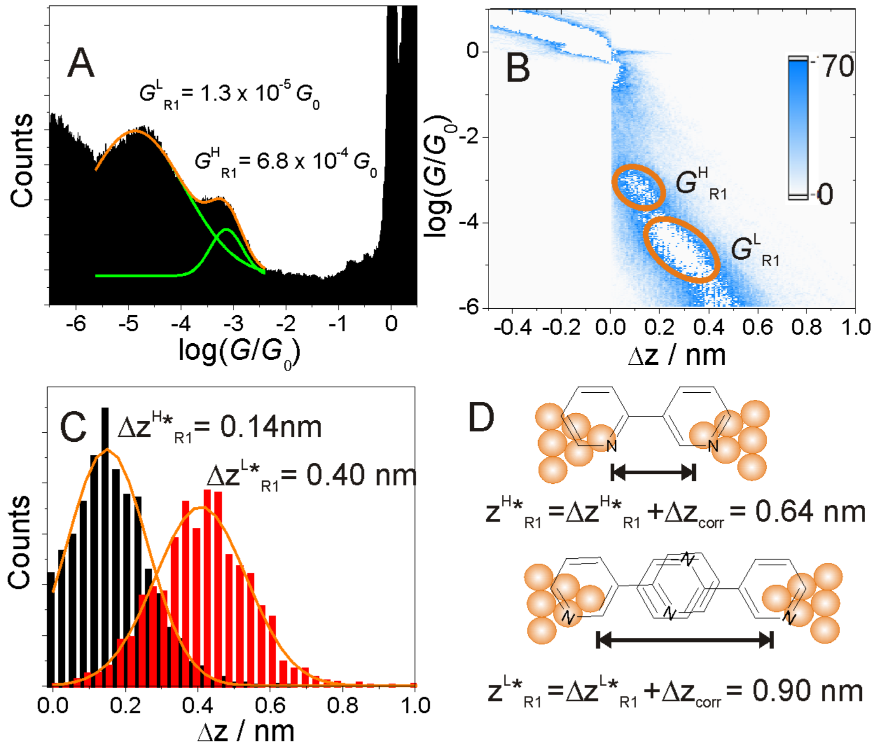

Figure S2: (A) 1D conductance histogram of **R1** molecule recorded at 100 mV bias. (B) 2D histogram generated from more than 1000 individual curves. (C) Characteristic length histograms analysed until high (H) and Low (L) conductance ranges. (D) Schematics of possible molecular junction configurations for high (H) and Low (L) conductances observed in 1D and 2D histograms with most probable electrode separation distances.

Conductance measurements of individual molecular units (R1 and R2): We measured the conductance of **R1** and **R2** molecules under similar conditions used for **TP1**. **R1** molecule showed two distinct conductance features (High (H) and Low (L)) in 1D and 2D conductance

histograms (Supplementary Fig. S2 (A,B)). Based on the characteristic lengths extracted from Fig. S2C, we assigned the most probable junction configurations for high (H) and Low (L) conductance features in Fig. S2D. Molecule **R2** showed only one clear conductance peak in the histograms and schematic of corresponding junction configuration was shown in Fig. S3.

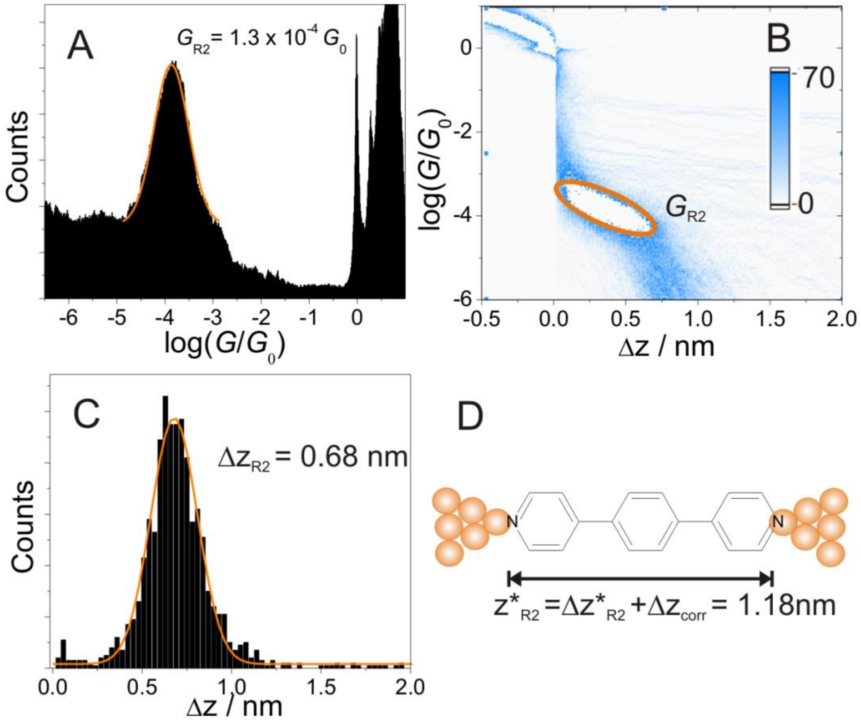

Figure S3: (A) 1D conductance histogram of **R2** molecule recorded at 100 mV bias. (B) 2D histogram generated from more than 1000 individual curves. (C) Characteristic length histograms analysed until high (H) and Low (L) conductance ranges. (D) Schematics of possible molecular junction configuration and the most probable electrode separation distance.

Molecule	Conductance	G/G_0	Δz_i * / nm	$z_i^* = \Delta z_i^* + \Delta z_{corr}$ / nm
**TP1**	G_1	6.3×10^{-3}	≈0.05-0.15	≈0.45-0.65
	G_2	3.6×10^{-4}	0.42	0.92 ± 0.1
	G_3	1.1×10^{-5}	0.86	1.36 ± 0.1
	G_4	1.8×10^{-7}	1.21	1.71 ± 0.1
**R1**	G^H_{R1}	6.8×10^{-4}	0.14	0.64 ± 0.1
	G^L_{R1}	1.3×10^{-5}	0.40	0.90 ± 0.1
**R2**	G_{R2}	1.3×10^{-4}	0.68	1.18 ± 0.1

Table.S1: Summary of characteristic lengths Δz* and the conductance values the investigated molecules.

S.3 Geometry optimizations and conformational sampling for TP1

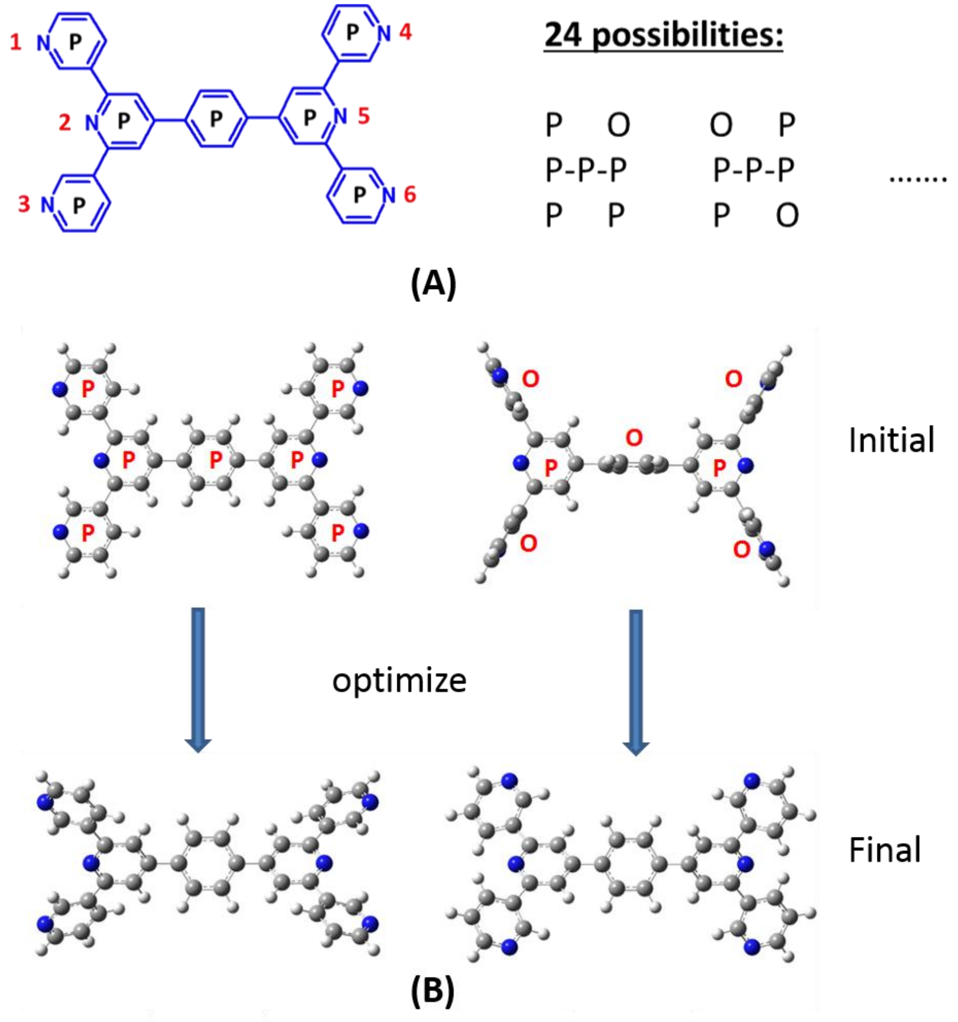

Figure S4: Procedure to generate multiple geometries which account for the torsional flexibility of **TP1**.

As the molecule is expected to exhibit significant conformational flexibility in terms of ring rotations, we considered multiple geometries of **TP1** which differ in terms of their relative orientations of the pyridine rings in our calculations. Different conformations of **TP1** were manually drawn wherein each aromatic ring was allowed to adopt one of two conformations, either in plane (P) or orthogonal (O) to, relative to its neighboring rings (Fig. S4A). This procedure resulting in 24 distinct starting geometries, each of which were optimized in Gaussian 09 using DFT with a B3LYP exchange correlation functional and a 6-31G* basis set.[9] The optimization yielded 18 unique geometries. Examples of two optimized geometries starting from

an all-planar (adjacent rings in plane) configuration and from an all-orthogonal (plane adjacent rings orthogonal to each other). Free energies for each optimized geometry were calculated using thermochemical analysis within Gaussian. Based on the small free energy differences all 18 geometries are thermodynamically accessible at room temperature. The inset in Fig. S5A shows

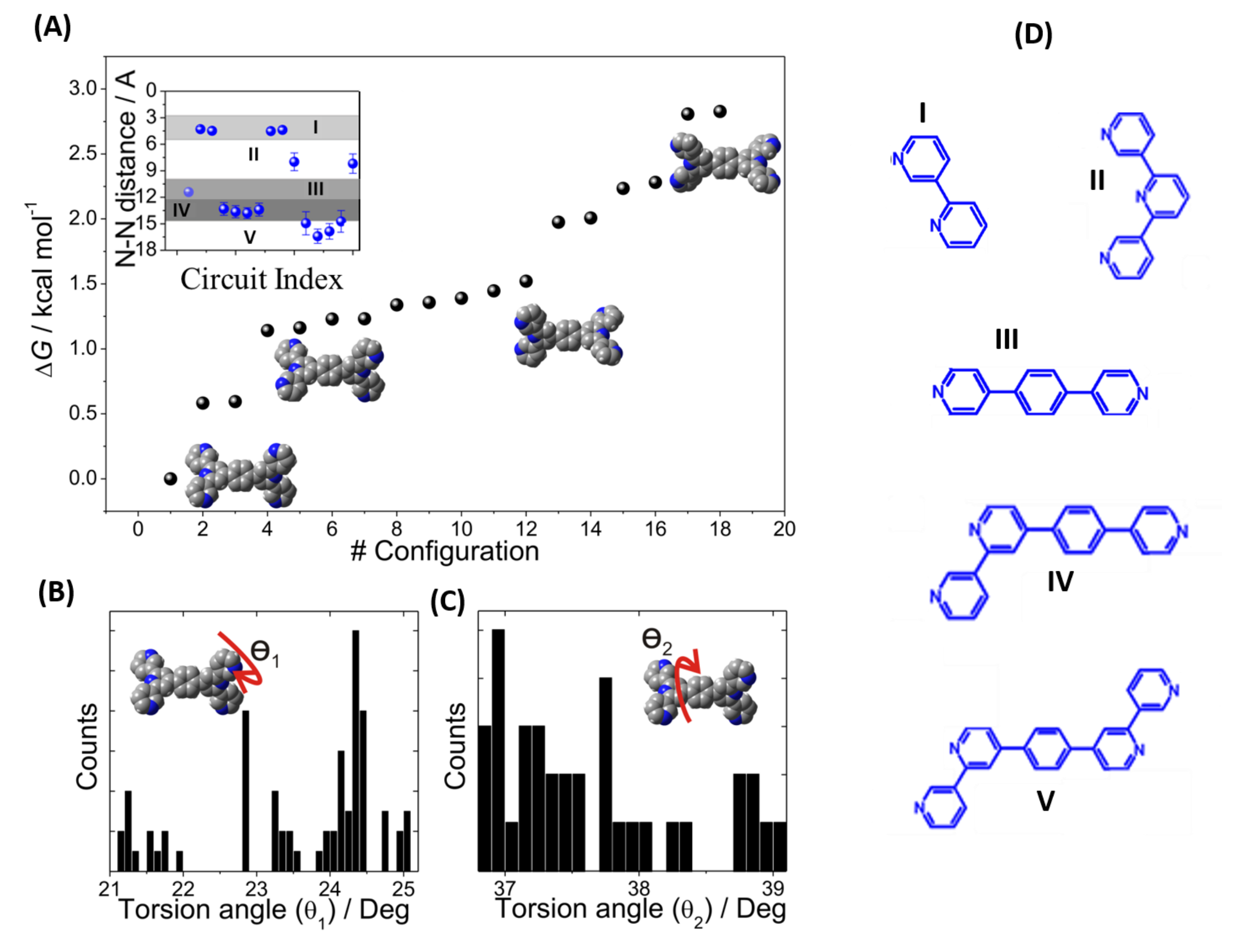

Figure S5: Effect of Conformational Flexibility on Electronic Structure: (A) Relative energies of 18 unique conformations of **TP1** obtained from geometry optimizations carried out on 24 different starting geometries which differ in the relative orientation of the seven aromatic rings of **TP1** (see methods and section S.3 of SI). The energies cluster into four classes which correspond to non-planar geometries with terminal nitrogens on each terpyridine arm either pointing towards or away from each other. Representative conformations for each of the four classes are shown. The inset shows variation of the 15 nitrogen pair distances in **TP1** over all 18 optimized conformations. (B) Distribution of torsion angles between adjacent pyridine rings within the terpyridine arms. (C) Distribution of dihederal angles between pyridine and benzene rings.(D) Single terminal circuits corresponding to the bands of N-N distances shown in the inset of panel (A).

the variation of the 15 nitrogen pair distances in **TP1** across the 18 optimized molecular geometries. Four distinct groupings appear (shaded in grey). These correspond to (Fig. S5D) 2-

ring circuits within each terpyridine arm (I), 3-ring circuits across each terpyrdine arm (II), 3-ring circuits in the TP1 core (III), 4-ring circuits spanning the core and one of the terpyridine arms (IV), and 5-ring circuits spanning both terpyridine arms of **TP1** (V). Circuits II and V show the largest fluctuations in terminal N-N distances. The terminal N-N distance for circuit III does not vary at all across the 18 optimized geometries.

S.4 Enumeration of TP1 circuits In the result section we presented an enumeration of **TP1** circuits. Here we elaborate upon this enumeration in more detail. Essentially we have a total of 6

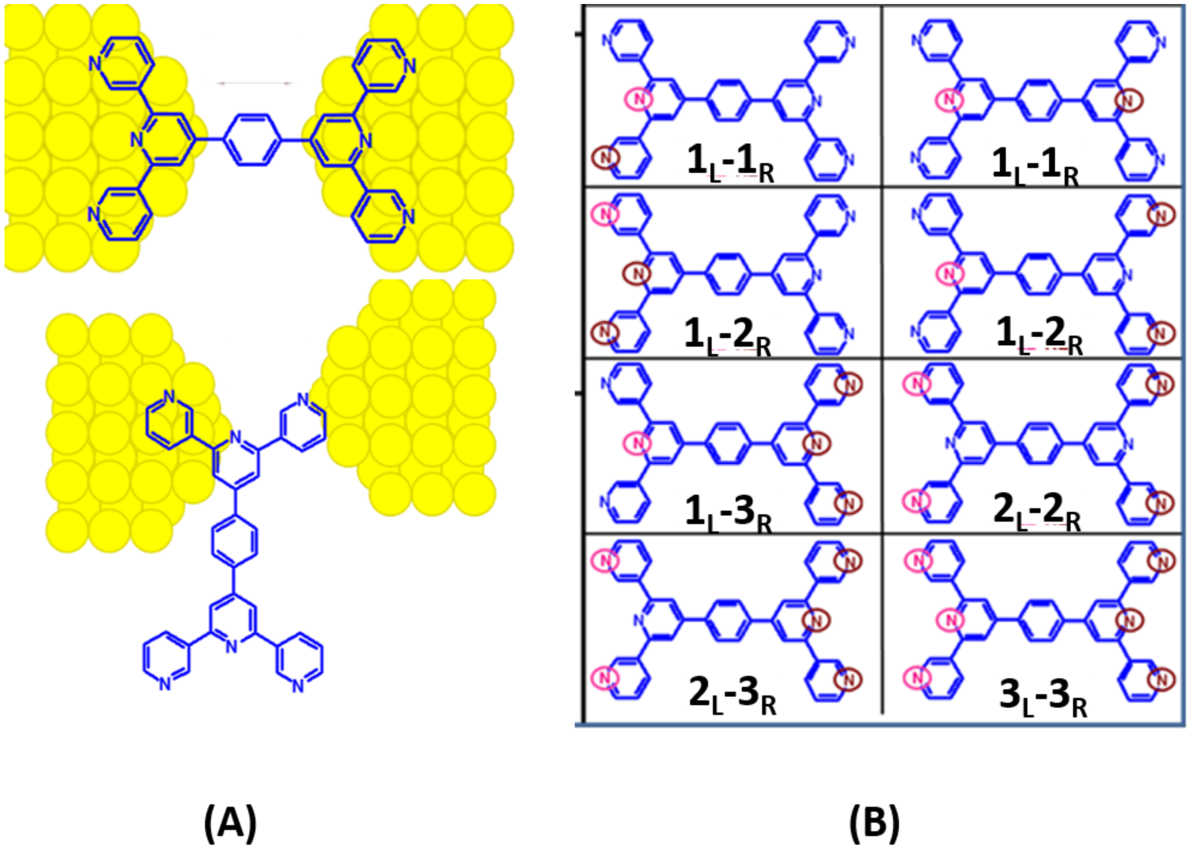

Figure S6: Examples of the possible modes of contact for the **TP1** molecular breadboard. We assume only two electrode circuits, wherein each electrode can make contact with upto three atoms within a terpyridine arms or across the terpyridine atoms (A). Some examples of single and multi-terminal circuits for the **TP1** molecular breadboard are also shown (B).

nitrogen positions, three on the left terpyridine arm (C_1,C_2,C_3) and three on the right terpyridine arm (C_1',C_2',C_3') at which the molecule can be anchored to the electrodes. We consider only two electrode circuits wherein each electrode contacts distinct sets of atoms either within each terpyridine arm or across two terpyridine arms (Fig. S6A)). If we assume that each electrode can

contact between 1-5 nitrogen atoms a total of 301 circuits can be generated. These comprise $C_1^6 \times C_1^5/2 = 15$ (1_L-1_R), $C_2^6 \times C_2^4/2 = 45$ (2_L-2_R), $C_2^6 \times C_1^4 = 60$ (2_R-1_R/1_L-2_R), $C_3^6 \times C_3^3/2 = 10$ (3_L-3_R), $C_3^6 \times C_2^3 = 60$ (3_L-2_R/2_L-3_R), $C_3^6 \times C_1^3 = 60$ (3_L-1_R/ 1_L-3_R), $C_4^6 \times C_2^2 = 15$ (4_L-2_R/2_L-4_R), $C_4^6 \times C_1^2 = 30$ (4_L-1_R/1_L-4_R), and $C_5^6 \times C_1^1 = 6$ (5_L-1_R/1_L-5_R) contact configurations. Here we express contact configurations in terms of the number of contacts (M, N) made by the two (L/R) electrodes as M_L-N_R, (where M,N=1,2,3). However, not all these configurations can be accessed within a break-junction setup. If we apply the more conservative constraint that a single electrode cannot contact both terpyridine arms simultaenously i.e each electrode can contact upto a maximum of three nitrogen atoms which lie within a single terpyrdine arm. Based on the above assumptions each electrode can contact seven combinations of atoms belonging to either the right (C_1, C_2, C_3, C_1+C_2, C_2+C_3, C_1+C_3, C_1+C_2+ C_3) or the left (C_1', C_2', C_3', C_1'+C_2', C_2'+C_3', C_1'+C_3', C_1'+C_2'+ C_3') terpyrdine arms. These assumptions lead to the enumeration provided in the main manuscript in Table 1. Examples of these circuits are shown in Fig. S6.

S.5 NEGF Transport Calculations

Within the NEGF framework, [10] the molecular junction is partitioned into three subsystems (Fig. 1): a device region (the isolated molecule) and two structure-less electrodes. The Green's function describes the molecule and its interactions with the electrodes:

$$G(E) = \frac{1}{(E\mathbf{I} - \mathbf{H} - \Sigma^L - \Sigma^R)} \tag{S1}$$

H is the Hamiltonian of the isolated molecule provided by the INDO/s calculations. The self-energies Σ describe the broadening and shifts in molecular energies induced by the right (R) and left (L) electrodes. The transmission coefficient sums over all pathways for charge transport at energy E from one electrode to the other:

$$\mathcal{T}_{LR}(E) = Tr(\Gamma^L G \Gamma^R G^\dagger) \tag{S2}$$

The broadening matrix: $\Gamma = i\,[\Sigma - \Sigma^+]$ is proportional to the imaginary part of the self-energy. We assumed that the **TP1** molecule is connected to the electrodes through the nitrogen atoms of the flanking terpyridine arms (Fig. 1A). We adopt the weak coupling limit, discussed extensively in Xing et. al.,[11] wherein the electrode atoms are not explicitly modelled. Instead, the effect of the electrodes is introduced through the broadening matrix as a parameter represented in our calculations in the basis of atomic orbitals. The molecule is assumed to contact the metal

electrodes only via the nitrogen atoms, i.e. only orbitals of the nitrogen atoms are broadened and the couplings among atomic orbitals are not affected by the contacts. The elements of the broadening matrix, are:

$$\Gamma_{ii} = \Gamma_N \quad \text{for all nitrogen atoms atomic orbitals}$$
$$= 0 \quad \text{otherwise}$$
$$\Gamma_{ij} = 0 \quad \text{for } i \neq j \tag{S3}$$

Here, i and j are atomic orbital indices and Γ_N is the nitrogen-gold coupling parameter set to 0.1 eV. We note that all nitrogen atoms are not equally accessible to electrode and assume a molecule-electrode model described in section S.7 below. We neglect the real part of the self-energy in our calculations. In the linear response regime, the conductance is given by the Landauer expression:

$$G = \frac{2q}{hV} \int \mathcal{T}_{LR}(E)[f_L(E) - f_R(E)]dE \tag{S4}$$

The Fermi functions $f_{L/R}$ define the electron occupancy based on the chemical potentials $\mu_{L/R}$ (Fig. 1) of left and right electrode:

$$f_{L/R}(E) = \frac{1}{1 + \exp[(E - \mu_{L/R})/k_B T]} \tag{S5}$$

Where k_B is the Boltzmann constant and temperature T=300 K. For tunneling charge transport, the conductance is dominated by the contribution at the Fermi energy, and we compute the tunneling conductance:

$$G_{Tunnel}(E) \sim \frac{2q^2}{h} \mathcal{T}_{LR}(E = E_F) \tag{S6}$$

The Fermi energy E_F is a free parameter set to different values as described in the methods section of the main manuscript and section S.9 below. V is the applied potential bias, taken as 100 mV in the calculations here.

S.6.Decomposition of multi-terminal currents into single terminal currents

The computed conductance for the different circuit within the **TP1** breadboard in Fig. 4 of the main manuscript spans 5 orders of magnitude and is clustered into three conductance bands. The distribution of conductance values in the different conductance appears to be not sensitive to the number of terminal (contacts) made by the molecule to the electrodes. For instance single terminal (1_L-1_R) circuits and multi-terminal (M_L-N_R; where M=2,3 and/or N=2,3) circuits both

span the entire range of conductance values. Here we rationalize these observations by showing

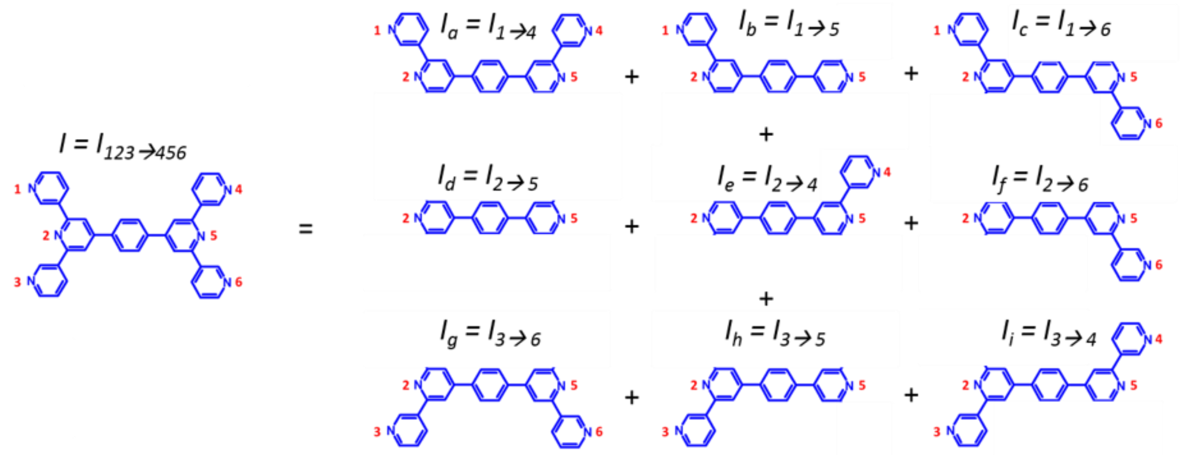

Figure S7: Example of the decomposition of a multi-terminal circuit into constituent single terminal circuits. The current through the multi-terminal circuit 3_L-3_R on the left can be expressed as the sum of currents flowing through the nine constituent single terminal 3-,4-, and 5-ring circuits on the right. For this particular example, the current through the 3-ring circuit I_d is orders of magnitude larger than that through the other eight circuits, fully determining the multi-terminal circuit current.

formally that the conductance of multi-terminal circuits can be decomposed into constituent single terminal circuits. Consider a circuit (Fig. S7) with left (right) electrode contacting all three nitrogens on the left (right) terpyridine arm of **TP1** with current $I_{123\to456}$ (V). This circuit can be decomposed into nine single terminal parallel circuits (Fig. S7) with currents $I_{n\to m}\,(V)\;\; where\;\; n = 1,2,3; m = 4,5,6.$ We now show that the Kirchoff`s parallel circuit rule will apply in general for circuit decompositions of the type shown in Fig. S7 for the **TP1** molecular breadboard. Using eqns S2 and S6, we write the near zero bias current in any general circuit within a molecular breadboard containing N atoms as:

$$I_{L\to R} = \frac{2q^2}{h} Tr[\Gamma^L(1,2..n)G(E = E_F)\Gamma^R(1',2'\dots m)G^\dagger(E = E_F)] \qquad (S7)$$

Where, we assume that the left and right electrode contact n, and m sets of distinct atoms (n+$m \leq$ N). The N×N broadening matrices $\Gamma^{L/R}(a,b,c,\dots)$ are diagonal with non-zero elements at indices corresponding to the contacted atoms a,b,c (see eqn. 3) of the breadboard. Decomposing the broadening matrices in terms of single atom contacts ($\Gamma^{L/R}(a,b,c,\dots) = \Gamma^{L/R}(a) + \Gamma^{L/R}(b) + \Gamma^{L/R}(c)$ ....), we write:

$$I_{L\to R} = \frac{2q^2}{h} Tr\left[\left(\sum_{k=1}^{n}\Gamma^L(k)\right)G(E = E_F)\left(\sum_{l=1'}^{m}\Gamma^R(l)\right)G^\dagger(E = E_F)\right] \qquad (S8)$$

Here the trace is carried out in the atomic basis where the matrices of Green`s functions are not diagonal. Explicit evaluation of the trace yields.

$$I_{L\rightarrow R} = \frac{2q^2}{h}\left(\sum_{k=1}^{n}\sum_{l=1'}^{m}\left[\Gamma_{kk}^{L}G_{kl}\Gamma_{ll}^{R}G_{lk}^{\dagger}\right]\right) = \frac{2q^2}{h}\left(\sum_{k=1}^{n}\sum_{l=1'}^{m}Tr\left[\Gamma^{L}(k)G\Gamma^{R}(l)G^{\dagger}\right]\right) = \sum_{k=1}^{n}\sum_{l=1'}^{m}I_{k\rightarrow l} \quad \text{(S9)}$$

Thereby verifying Kirchoff`s rule for parallel circuits in molecular breadboards. Note that in deriving eqn. S9, we assumed conditions of a near zero bias tunneling current, where: 1) the current is dominated by the transmission at Fermi energy (eqn. S6), and 2) that the diagonal terms of the Green`s function satisfy: $(E_F - H - \sum(\Gamma^L + \Gamma^R))^{-1} \approx (E_F - H)^{-1}$. Both conditions are satisfied when the tunneling barrier for tunneling is large relative to the broadening of the molecular electronic energies introduced by the electrodes. The conductance data in Fig. 4 for multi-terminal and single-terminal circuits can be thus be rationalized under these conditions wherein all multi-terminal circuits within the breadboard can be decomposed into constituent single-terminal circuits. If one constituent single terminal current is dominant (much larger than other currents), the conductance from the multi-terminal circuit will be totally determined by that single channel circuit as observed for the top two conductance bands in Fig. 4. For the specific example in Fig. S7, the current I_d of the single terminal core ring circuit is the dominant current fully determining the conductance of the multi-terminal 3_L-3_R circuit.

S.7 Molecule-Electrode coupling model

In order to account for the different electrode accessibility of nitrogen atoms in **TP1**, we assumed a molecule-electrode coupling model wherein the broadening matrix includes different electrode electronic couplings for peripheral (end pyridine rings) and core (central pyridine rings) nitrogen atoms on the terpyridine units of **TP1**:

Γ_{ii} = $\Gamma_{Peripheral}$ for nitrogen atoms on terminal pyridine rings of the terpyridine units

= Γ_{Core} for nitrogen atoms on central pyridine rings of the terpyrdine units

= 0 otherwise

Γ_{ij} = 0 for i≠j (S10)

We assumed a molecule electrode electronic coupling decays exponentially with respect to

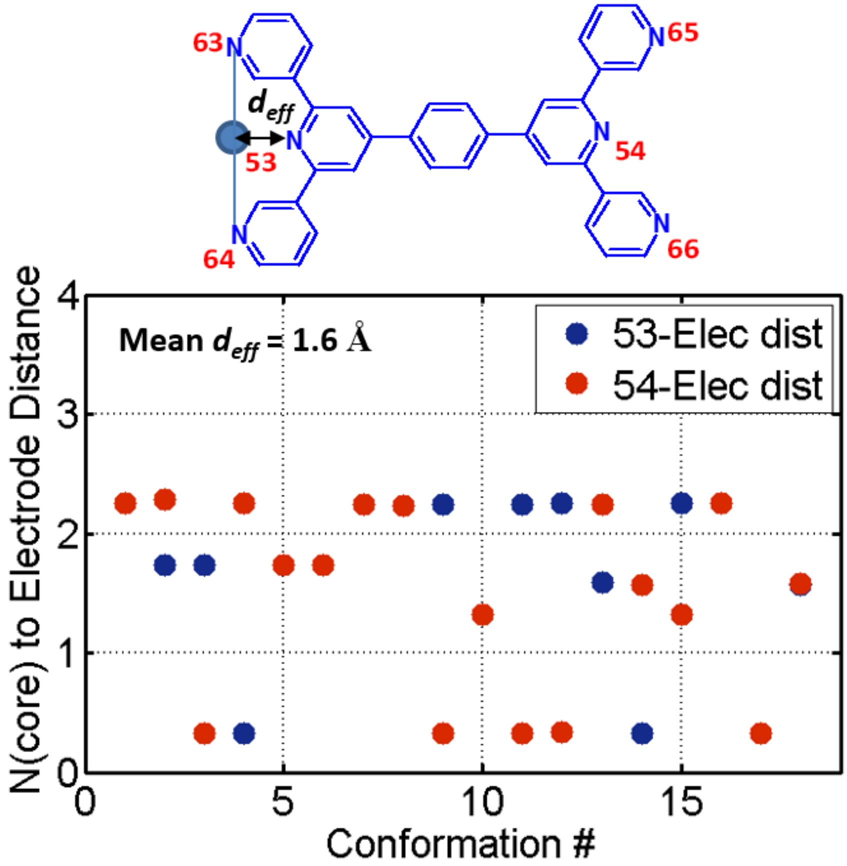

Figure S8: Examples of the possible modes of contact for the TP1 molecular breadboard. We assume only two electrode circuits, wherein each electrode can make contact with upto three atoms within a terpyridine arms or across the terpyridine atoms (panel A). Some examples of single and multiterminal circuits for the TP1 molecular breadboard are also

distance of each nitrogen atom. Thus, relative to the peripheral atoms, the central nitrogen atoms of each terpyridine arm should be further away from the electrode tips leading to a screening of electronic couplings between core nitrogen atoms and the electrode. We thus assumed $\Gamma_{peripheral}$ / Γ_{core} = $exp(\beta * d_{eff})$, where β=3.0 $Å^{-1}$ is the decay of the electronic coupling through vacuum. The effective screening length (d_{eff}) was defined as the distance between the central nitrogen atom and the centre of mass of the peripheral nitrogen atoms for each terpyridine arm of **TP1** (Fig. S8). We estimated d_{eff} ~ 1.6 *Å* (Fig. S8), leading to an electronic coupling attenuation ratio $\Gamma_{peripheral}$ /Γ_{core} = 116**.**

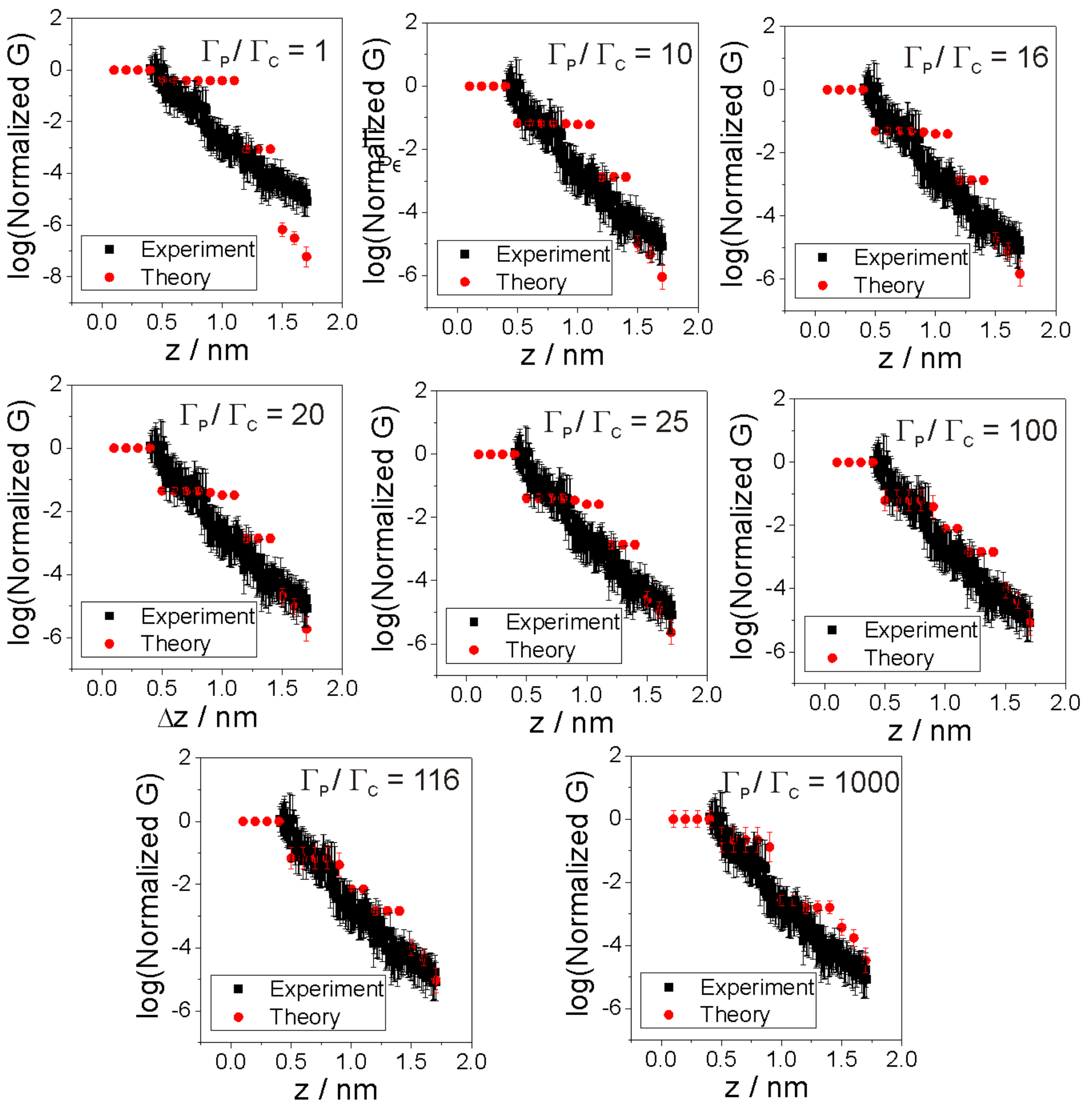

Figure S9: Comparison 2-D plots of computed total conductance from 61 circuits (red symbols) for hole mediated transport (E_F – E_{HOMO} ~ 2.9 eV) and master curve extracted from 2D-histogram of MCBJ measurements (black symbols). The contributions of each of the 61 circuits to the computed total conductance was averaged over 18 optimized **TP1** geometries. Since our computations only capture relative trends, both computed and experimental conductances were normalized with respect to the conductance values at the smallest electrode separation. Error bars for the computed

Independently, we carried out conductance calculations varying $\Gamma_{peripheral}$ /Γ_{core} over three orders of magnitude. For each ratio, the conductance values for all 61 different circuits were computed at the 18 different optimized geometries to generate conductance data as a function of electrode separation as in Fig. 5. Comparison plots of the average total current computed over the 18 different optimized geometries and standard deviations at four different electrode separations

with the corresponding average conductance values extracted from the MCBJ experiments are shown in Fig. S9. We varied the ratio of peripheral to core nitrogen electrode coupling parameters $\Gamma_{Peripheral}/\Gamma_{Core}$ to find the optimal fit to the experimental results. The data in Fig. S9 shows that the fits are sensitive to the ratio varied over two orders magnitude and the best fits were obtained for $\Gamma_{Peripheral}$ / Γ_{Core} ~100. This independently corroborates the electronic coupling screening estimate extracted from the analysis of **TP1** structures and the fit reported in the main manuscript is for $\Gamma_{Peripheral}$ / Γ_{Core} ~116 (the value obtained from our structural analysis).

S.8 Electron vs hole dominated transport regimes for the TP1 breadboard

In the NEGF framework outlined above to compute conductance for the **TP1** breadboard, the Green`s function in eqn. S1 is computed from the electronic structure of **TP1** in isolation, while the effect of the electrodes is incorporated phenomenologically through the broadening function. In this framework, the Fermi energy E_F of the electrode is a variable parameter which can be set to a suitable value lying within the band gap of the organic system (**TP1**) unde

r investigation. There are a couple of different ways of setting a value for parameter E_F: 1) One can set E_F equal to the work function of the metal which forms the electrode material (for gold E_F = -5.1 eV), or 2) choose the value of E_F to match experimentally estimated electron or hole injection barriers. If the absolute values of electronic state energies (orbital energy level alignments in a one-electron picture) obtained from electronic structure theory are reliable, in the weak coupling limit, the difference between the electrode workfunction and the HOMO/LUMO energies will give reasonable estimates of hole/electron injection barriers. However, typically both occupied and unoccupied orbital energy level positions computed from electronic structure theory can be significantly offset from experimental values[12] creating uncertainties in the placement of the electrode Fermi level in molecular HOMO-LUMO gap. We have previously carried out the dependence of transport properties in different organic systems as a function of the Fermi level placement for several different organic molecules [11, 13-15] These studies show that for charge transport in the deep tunneling regime (hole/electron injection barriers > 1 eV and small applied biases of ~ 100 mV), properties such as relative conductance trends for molecules within the same class are insensitive to the choice of E_F. Since the conductance features of the **TP1** breadboard discussed in our present study depends crucially on relative conductance values of the different circuits within the breadboard, we expect that our observations in our previous

studies holds for the **TP1** breadboard studied here as well. Nevertheless, we still carried out full transport calculations for two different choices of E_F values as described below.

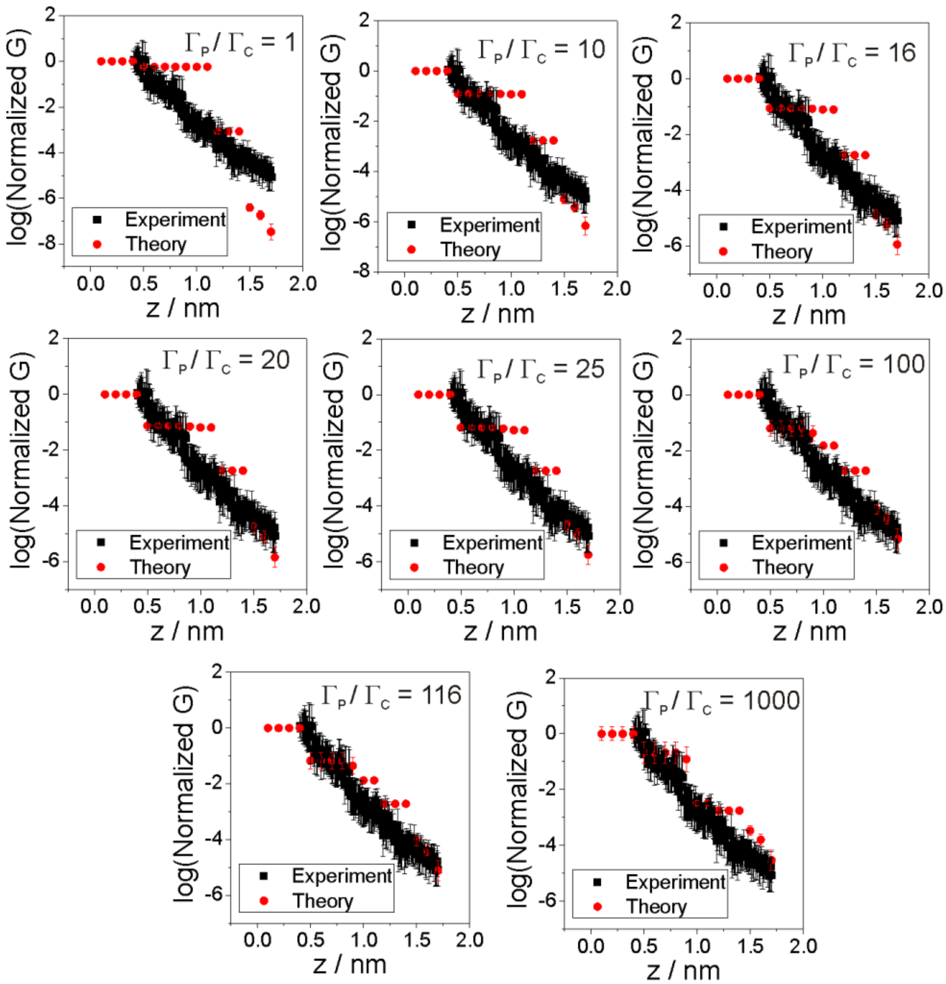

Figure S10: Same calculation as in Fig. S9 but for electron mediated transport ($E_{LUMO} - E_F \sim$ 2.9 eV).

For the INDO/s band gaps calculated for **TP1** geometries, the electrode Fermi level (set to -5.1 eV) lies closer to the HOMO (hole transport barrier $E_F - E_{HOMO} \sim$ 2.9 eV) than the LUMO. DFT calculated band gaps are lower with the electrode Fermi level again lying closer to the HOMO (hole transport barrier of $E_F - E_{HOMO} \sim$ 1.4 eV) than the LUMO. However, recent experiments on bis-terpyridine poly-p-phenyl molecules and molecules with pyridyl anchors have suggested that transport in these systems is in fact electron dominated.[16, 17] Thus, we also carried out calculations in the electron dominated regime by setting the Fermi level to lie 2.9 eV below the LUMO. For each E_F chosen, the conductance values for all 61 different circuits were

computed at the 18 different optimized geometries to generate conductance data as a function of electrode separation as in Fig. 6 (data shown for hole dominated transport; $E_F - E_{HOMO}$ =2.9 eV). Overall, the results presented in the main manuscript (Figs. 6) were found to be insensitive to the variation in E_F from electron to hole dominated regime. Here we present our best fits to the experimental data utilizing the restricted nitrogen accessibility model for molecule electrode coupling described in section S.7. Comparison plots of the average current over the 18 different optimized geometries and standard deviations at four different electrode separations with the corresponding average conductance values extracted from the MCBJ experiments are shown in Fig. S9 for hole dominated transport ($E_{HOMO} - E_F$ =2.9 eV) and Fig. S10 for hole dominated transport ($E_{LUMO} - E_F$ =2.9 eV). We varied the ratio of peripheral to core nitrogen electrode coupling parameters $\Gamma_{Peripheral}/\Gamma_{Core}$ to find the optimal fit to the experimental results. The fits for electron dominated regime calculations (Fig. S10) do not alter any of the conclusions drawn from the hole dominated regime calculations and fits (Fig. S9 and Figs. 4B and 5 in main manuscript). The data in S10 show that the fits are sensitive to the ratio $\Gamma_{Peripheral}$ / Γ_{Core} varied over two orders magnitude and the best fits were obtained for $\Gamma_{Peripheral}$ / Γ_{Core} =100.

S.9 Experimental and Computational Tunneling decay constants for the TP1 breadboard

In this section, we estimate the tunneling decay constant (β) for the **TP1** breadboard from theory and experimental data:

1) In figure S11A we have plotted the most probable conductance values and their standard-deviations for the G_1, G_2, G_3, and G_4 states extracted from Fig 3B as a function of electrode separation. The electrode separations were extracted from the peak positions of the characteristic length 1D distance histograms for the four conductance states G_1, G_2, G_3, and G_4 in Fig 3D. The electrode separation for the reference G_1 peak was set to the lower bound of the estimated snap-back distance value of 0.4 nm. By fitting the conductance data showed in Fig. 11A, we estimated conductance decay constant as $\beta_{Experiment}$ = 3.4 nm^{-1}.

2) From the computed conductance of the dominant 2-5 ring circuits assigned to the conductance plateaus in Fig 6. In figure S11B we plot the average conductance and standard-deviation for the 2-ring, meta-3-ring, para-3-ring, 4-ring, and 5-ring circuits at the corresponding electrode separations at which they dominate. By fitting the conductance data showed in Fig. 11B, we estimated conductance decay constant as β_{Theory} = 3.8 nm^{-1}.

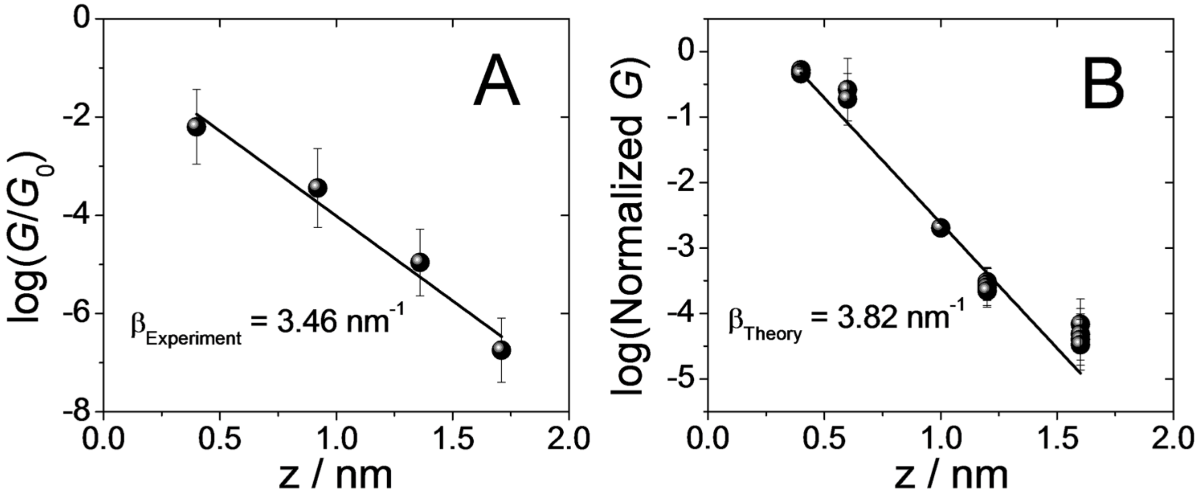

Figure S10: Plots of the conductance as a function of electrode separation (z) extracted from (A) the experimental data in Fig 3B and 3D, and from (B) the computed conductance of the dominant circuits assigned in Fig 6 to the conductance plateaus at different electrode separations. Theoretical circuit conductance values were calculated with $\Gamma_{Peripheral} / \Gamma_{Core}$ ~116 as in Fig 6.

S.10 Transmission features for the dominant circuits around the Fermi energy

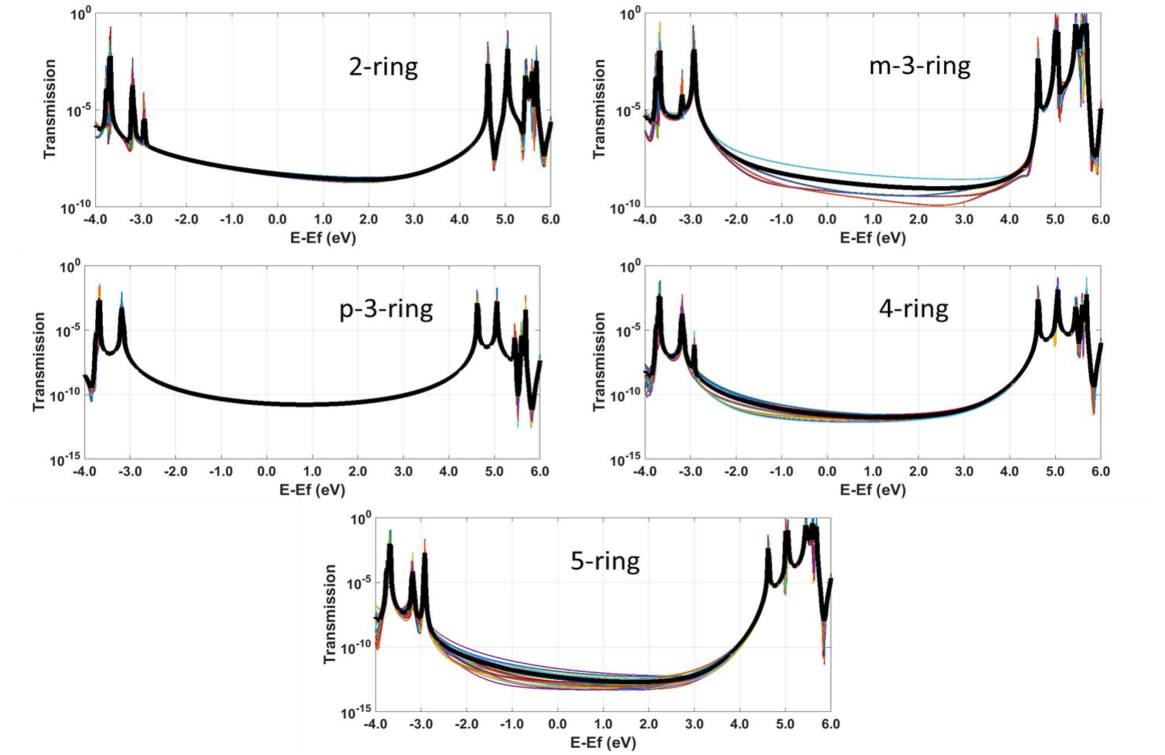

Figure S12: Transmission for the five dominant circuits assigned to conductance plateaus in Fig 6. Calculations were carried out for $\Gamma_{Peripheral}$ / Γ_{Core} ~116 to reflect the data in Fig 6.

REFERENCES

1. Yoshida, J.; Nishikiori, S.-I.; Yuge, H. *Journal of Coordination Chemistry* **2013,** 66, (12), 2191-2200.
2. Wang, J. H.; Hanan, G. S. *Synlett* **2005**, (8), 1251-1254.
3. Su, Y.-S.; Chen, C.-F. *Organic Letters* **2010,** 12, (8), 1888-1891.
4. Kudo, N.; Perseghini, M.; Fu, G. C. *Angew. Chem. Int. Ed.* **2006,** 45, (8), 1282-1284.
5. Kaliginedi, V.; Moreno-Garcia, P.; Valkenier, H.; Hong, W.; Garcia-Suarez, V. M.; Buiter, P.; Otten, J. L. H.; Hummelen, J. C.; Lambert, C. J.; Wandlowski, T. *J. Am. Chem. Soc.* **2012,** 134, (11), 5262-5275.
6. Hong, W.; Valkenier, H.; Meszaros, G.; Manrique, D. Z.; Mishchenko, A.; Putz, A.; Garcia, P. M.; Lambert, C. J.; Hummelen, J. C.; Wandlowski, T. *Beilstein Journal of Nanotechnology* **2011,** 2, 699-713.
7. Hong, W.; Manrique, D. Z.; Moreno-Garcia, P.; Gulcur, M.; Mishchenko, A.; Lambert, C. J.; Bryce, M. R.; Wandlowski, T. *J. Am. Chem. Soc.* **2012,** 134, (4), 2292-2304.
8. Moreno-Garcia, P.; Gulcur, M.; Manrique, D. Z.; Pope, T.; Hong, W.; Kaliginedi, V.; Huang, C.; Batsanov, A. S.; Bryce, M. R.; Lambert, C.; Wandlowski, T. *J. Am. Chem. Soc.* **2013,** 135, (33), 12228-12240.

9. M. J. Frisch; G. W. Trucks; H. B. Schlegel; G. E. Scuseria; M. A. Robb; J. R. Cheeseman; G. Scalmani; V. Barone; B. Mennucci; G. A. Petersson; H. Nakatsuji; M. Caricato; X. Li; H. P. Hratchian; A. F. Izmaylov; J. Bloino; G. Zheng; J. L. Sonnenberg; M. Hada; M. Ehara; K. Toyota; R. Fukuda; J. Hasegawa; M. Ishida; T. Nakajima; Y. Honda; O. Kitao; H. Nakai; T. Vreven; J. A. Montgomery, J.; J. E. Peralta; F. Ogliaro; M. Bearpark; J. J. Heyd; E. Brothers; K. N. Kudin; V. N. Staroverov; R. Kobayashi; J. Normand; K. Raghavachari; A. Rendell; J. C. Burant; S. S. Iyengar; J. Tomasi; M. Cossi; N. Rega; J. M. Millam; M. Klene; J. E. Knox; J. B. Cross; V. Bakken; C. Adamo; J. Jaramillo; R. Gomperts; R. E. Stratmann; O. Yazyev; A. J. Austin; R. Cammi; C. Pomelli; J. W. Ochterski; R. L. Martin; K. Morokuma; V. G. Zakrzewski; G. A. Voth; P. Salvador; J. J. Dannenberg; S. Dapprich; A. D. Daniels; Ö. Farkas; J. B. Foresman; J. V. Ortiz; J. Cioslowski; Fox, D. J. *Gaussian 09, Revision E.01*, Gaussian Inc: Wallingford CT, 2009.

10. Datta, S., *Quantum Transport: Atom to Transistor*. Cambridge University Press: 2005.

11. Xing, Y.; Park, T. H.; Venkatramani, R.; Keinan, S.; Beratan, D. N.; Therien, M. J.; Borguet, E. *J. Am. Chem. Soc.* **2010,** 132, (23), 7946-7956.

12. Zhang, G.; Musgrave, C. B. *J Phys Chem A* **2007,** 111, (8), 1554-1561.

13. Wierzbinski, E.; de Leon, A.; Yin, X.; Balaeff, A.; Davis, K. L.; Rapireddy, S.; Venkatramani, R.; Keinan, S.; Ly, D. H.; Madrid, M.; Beratan, D. N.; Achim, C.; Waldeck, D. H. *J. Am. Chem. Soc.* **2012,** 134, (31), 13141-13141.

14. Venkatramani, R.; Davis, K. L.; Wierzbinski, E.; Bezer, S.; Balaeff, A.; Keinan, S.; Paul, A.; Kocsis, L.; Beratan, D. N.; Achim, C.; Waldeck, D. H. *J. Am. Chem. Soc.* **2011,** 133, 62-72.

15. Paul, A.; Bezer, S.; Venkatramani, R.; Kocsis, L.; Wierzbinski, E.; Balaeff, A.; Keinan, S.; Beratan, D. N.; Achim, C.; Waldeck, D. H. *J. Am. Chem. Soc.* **2009,** 131, (18), 6498-6507.

16. Wang, W. H.; Wang, S. Y.; Li, X. Y.; Collin, J. P.; Liu, J.; Liu, P. N.; Lin, N. *J. Am. Chem. Soc.* **2010,** 132, (25), 8774-8778.

17. Widawsky, J. R.; Darancet, P.; Neaton, J. B.; Venkataraman, L. *Nano Lett.* **2012,** 12, (1), 354-358.
